# The Converging Effects of Different Categories of Antidepressants on the Brain: A Systematic Meta‐Analysis of Public Transcriptional Profiling Data From the Hippocampus and Cortex

**DOI:** 10.1111/jnc.70502

**Published:** 2026-07-03

**Authors:** Eva M. Geoghegan, Megan H. Hagenauer, Erin Hernandez, Sophia Espinoza, Elizabeth I. Flandreau, Phi T. Nguyen, Adrienne N. Santiago, Mubashshir Ra’eed Bhuiyan, Sophie Mensch, Stanley J. Watson, Huda Akil, René Hen

**Affiliations:** ^1^ Columbia University New York New York USA; ^2^ University of Michigan Ann Arbor Michigan USA; ^3^ University of Chicago Chicago Illinois USA; ^4^ Michigan State University East Lansing Michigan USA; ^5^ Grand Valley State University Allendale Michigan USA

**Keywords:** antidepressant, hippocampus, meta‐analysis, RNA‐seq

## Abstract

Depression can be treated with traditional pharmaceuticals targeting monoaminergic function, nontraditional drug classes and neuromodulatory interventions. To identify mechanisms of action shared across clinically‐effective antidepressant treatment categories, we performed two systematic meta‐analyses of public transcriptional profiling data from adult laboratory rodents (rats, mice). The outcome variable was gene expression, measured by microarray or RNA‐Seq from bulk‐dissected tissue from two depression‐related brain regions (hippocampus, cortex). Relevant datasets were identified in the Gemma database of curated, reprocessed transcriptional profiling data using predefined search terms and inclusion/exclusion criteria (*hippocampus*: June 24, 2024, *cortex*: July 10, 2024). Differential expression results were extracted for all genes, minimizing bias. For each gene, a random effects meta‐analysis model was fit to antidepressant vs. control effect sizes (Log2 Fold Changes) from each study for each brain region, with follow‐up analyses exploring sources of effect heterogeneity. For the hippocampus, 15 relevant studies were identified, containing 22 antidepressant vs. control group comparisons (collective *n* = 313 samples), with approximately half representing traditional versus nontraditional antidepressants. Of 16 439 analyzed genes, 58 were consistently differentially expressed (False Discovery Rate (FDR) < 0.05) following treatment. Antidepressant effects were enriched in the dentate gyrus and in gene sets related to stress regulation, brain growth and plasticity, vasculature and glia, and immune function. Comparisons with single nucleus RNA‐Seq confirmed effects on specific hippocampal cell types, including potential rejuvenation of dentate granule neurons. For the cortex, 13 studies were identified, containing 16 antidepressant vs. control group comparisons (collective *n* = 233 samples). Of 15 583 analyzed genes, only one was consistently differentially expressed (FDR < 0.05: *Atp6v1b2*), but overall expression patterns moderately resembled the hippocampus. These genes and pathways showing consistent differential expression across treatment categories may be promising targets for novel therapies. Future work should explore relevance to human clinical populations and potential heterogeneity introduced by sex and subregion.

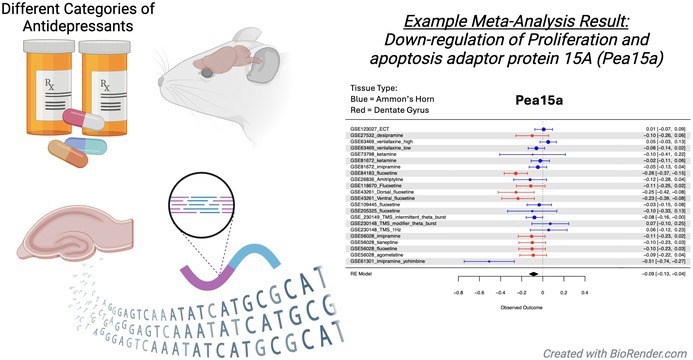

## Graphical abstract

Understanding the congruent effects of different types of antidepressant treatments on sensitive brain regions, such as the hippocampus and cortex, can highlight essential mechanisms of action. We used a systematic meta‐analysis of public transcriptional profiling datasets to identify genes and functional gene sets that are differentially expressed across antidepressant categories.

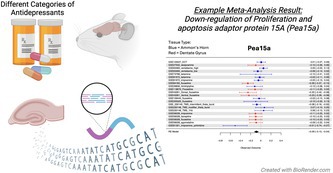

**Abbreviations**
ACG: anterior cingulate cortex
AHN: adult hippocampal neurogenesis
API: application programming interface
BDNF: brain‐derived neurotrophic factor
CA: cornu ammonis
CA1, CA2, CA3: cornu ammonis regions 1, 2, and 3
CI_lb: confidence interval lower bound
CI_ub: confidence interval upper bound
Cook's *D*
: Cook's distance
CSDS: chronic social defeat stress
Ctrl: control
CTX: cortex
CUMS: chronic unpredictable mild stress
d: day
DBS: deep brain stimulation
DEG: differentially expressed gene
df: degrees of freedom
DFBetas: differences in betas (indicator of influential observations)
DG: dentate gyrus
DLPFC: dorsolateral prefrontal cortex
DNA: (deoxyribonucleic acid)
ECS: electroconvulsive shock
ECT: electroconvulsive therapy
FDR: false discovery rate
fGSEA: fast gene set enrichment analysis
FLX: fluoxetine
GABA: Gamma‐Aminobutyric Acid
*H* ^2^
: estimated residual variance in effect size, as a ratio compared to total variability
HPC: Hippocampus
*I* ^2^
: estimated residual variance in effect size, as a percent of total variability
Log2FC: log(2) fold change
LPS: lipopolysaccharide (LPS)‐induced depression‐like model
MAOI: monoamine oxidase inhibitor
MASSA: melatonin agonist and selective serotonin antagonist
MDD: Major Depressive Disorder
MDMA: 3,4‐Methylenedioxymethamphetamine
mRNA: messenger RNA
*n*
: sample size
NMDAR: N‐Methyl‐D‐aspartate receptor
OBX: olfactory bulbectomy
*p*
: *p*‐value
PFC: prefrontal cortex
PRISMA: Preferred Reporting Items for Systematic reviews and Meta‐Analyses
*R*
: Pearson correlation coefficient
RE Model: random effects model
*Rho*
: Spearman correlation coefficient
RNA: ribonucleic acid
RNA‐Seq: RNA‐Sequencing
ROI: region of interest
SE: standard error
SIA: stress‐induced analgesia
SNDRA: serotonin–norepinephrine–dopamine releasing agent
SNRI: serotonin‐norepinephrine reuptake inhibitor
snRNA‐Seq: single nuclei RNA‐Sequencing
SSRI: selective serotonin reuptake inhibitor
*T*
: T‐statistic
Tau^2^
: estimated residual variance in effect size
TCA: tricyclic antidepressant
TMS: transcranial magnetic stimulation
v: version
wk: week
*β*
: regression coefficient

## 1 Introduction

Major Depressive Disorder (MDD) is characterized by consistent depressed mood or markedly diminished pleasure in daily activities (American Psychiatric Association 2013). Worldwide, an estimated 5% of adults suffer from depression, making it a leading cause of disability (World Health Organization 2023; Friedrich 2017) and increased mortality (Kessler and Bromet 2013). The current standard of care includes evidence‐based psychotherapies and specific classes of drugs, which may be guided by blood biomarkers predictive of treatment outcomes (Cohen and DeRubeis 2018; Uher et al. 2014). Although antidepressant treatments are effective for some patients, around 30%–40% of patients fail to respond to first‐line treatment (Norman and Olver 2019).

To improve individualized treatment options, the mechanisms of action for different antidepressant treatments need to be investigated further to understand both their congruent and differing neurobiological effects. Many first‐line antidepressants enhance monoamine neurotransmitter function, such as the serotonergic, noradrenergic, and dopaminergic systems. These include selective serotonin reuptake inhibitors (SSRIs), serotonin‐norepinephrine reuptake inhibitors (SNRIs), tricyclic antidepressants (TCAs), and monoamine oxidase inhibitors (MAOIs) (Gautam et al. 2017; Harmer et al. 2017; Artigas et al. 2002). However, monoamine potentiation may not be the direct mechanism of action of these drugs, as monoamine potentiation occurs rapidly and clinical improvement usually requires several weeks (Harmer et al. 2017).

Other classes of pharmacological treatments have shown promise for treating depression. Ketamine, a N‐Methyl‐D‐aspartate receptor (NMDAR) antagonist, has rapid and long‐lasting antidepressant effects at subanesthetic doses (Zanos and Gould 2018; Homayoun and Moghaddam 2007; Carreno et al. 2016). Agomelatine has synergistic effects as an agonist of melatonin and some serotonin receptors (Norman and Olver 2019; Guardiola‐Lemaitre et al. 2014; Racagni et al. 2011). Tianeptine is an atypical tricyclic believed to have antidepressant effects via μ‐opioid receptor agonism (Samuels et al. 2017; Han et al. 2022). Other promising new classes include psychedelics (MDMA, psilocybin) (Gill et al. 2020) and second generation antipsychotics (e.g., quetiapine) (Tran and Argáez 2020). Finally, alpha‐2 adrenergic antagonists may accelerate TCA treatment response times by reversing treatment‐related alpha‐2 adrenoceptor desensitization (Invernizzi and Garattini 2004; Smith and Hollingsworth 1984). Depression is also treated using neuromodulatory brain stimulation therapies, such as electroconvulsive therapy (ECT), deep brain stimulation (DBS) and transcranial magnetic stimulation (TMS) (Figee et al. 2022; Kisely et al. 2018; Singh and Kar 2017; Deng et al. 2024; Downar et al. 2024; Sonmez et al. 2019). Altogether, the diversity of neurobiological pathways targeted by these new treatments suggests that monaminergic effects may not be central to antidepressant response, or may lie upstream of other critical pathways.

In addition to diverse molecular targets, there are multiple brain regions that may mediate antidepressant response. Antidepressant treatments are often theorized to alleviate depressive symptoms via effects on the hippocampus. Hippocampal volume reduction is common in patients with depression (Belleau et al. 2019). This atrophy may be due to prolonged exposure to stress hormones (glucocorticoids) and subsequent reduction in neurotrophic factors and adult hippocampal neurogenesis (AHN) in the dentate gyrus (DG) (Belleau et al. 2019), both of which precede mood disturbance (Roddy et al. 2019; Sheline 2011; Charney and Manji 2004). Antidepressants reverse or prevent hippocampal atrophy in depression. Growing evidence suggests that both traditional, monoaminergic‐targeting antidepressants (e.g., SSRIs, TCAs) (Sahay and Hen 2007; Santarelli et al. 2003) and other antidepressant treatments (e.g., ECT) (Madsen et al. 2000; Loef et al. 2023) reverse hippocampal atrophy by stopping stress‐induced dendritic retraction, increasing growth factor signaling (e.g., brain‐derived neurotrophic factor (BDNF) (Zanos and Gould 2018; Guardiola‐Lemaitre et al. 2014; Racagni et al. 2011; Duman et al. 2021), and increasing hippocampal neuroplasticity and AHN (Sahay and Hen 2007; Sapolsky 2001; Czéh et al. 2001)). However, it is still unclear whether increases in hippocampal neuroplasticity and AHN are a central mechanism for all antidepressant classes.

Some antidepressants may also target the cortex. Depressed patients have documented cortical abnormalities, including thinner cortical gray matter (Schmaal et al. 2017) and reduced prefrontal cortex (PFC) volume (Krishnan and Nestler 2011). Rodent models of depression show similar structural and physiological changes, including dendritic shrinkage and reductions in myelination in the PFC (Pizzagalli and Roberts 2022). Antidepressant treatments may reverse these changes by directly modulating cortical function. For example, TMS is thought to facilitate synaptic plasticity within the target site, which is most commonly the dorsolateral prefrontal cortex (DLPFC) (Downar et al. 2024), by increasing cortical activity (Rizvi and Khan 2019), and antidepressant DBS targets cortical white matter tracts (Figee et al. 2022). Antidepressant pharmaceuticals can also modulate cortical activity. As an NMDA receptor antagonist, ketamine decreases cortical GABAergic interneuron activity, causing downstream disinhibition (Homayoun and Moghaddam 2007) and increasing synaptic plasticity (Zanos and Gould 2018; Homayoun and Moghaddam 2007; Carreno et al. 2016). Similarly, monoaminergic antidepressants (SSRIs, SNRIs) have been shown to increase activity or metabolism in frontal cortical regions (Bellani et al. 2011). However, whether cortical plasticity is a central mechanism for all antidepressant classes remains unknown.

In order to better understand the effects of antidepressant treatments on the hippocampus and cortex, we conducted two systematic meta‐analyses of publicly available rodent transcriptional profiling data, as well as a variety of follow‐up exploratory analyses. Transcriptional profiling technologies, such as ribonucleic acid‐sequencing (RNA‐Seq) and microarray, quantify gene expression in tissues. By conducting meta‐analyses across studies using different classes of antidepressants, we hoped to identify patterns of gene expression common to a wide variety of antidepressant treatments while also increasing statistical power to provide stronger insights into gene expression patterns underlying functional changes.

## 2 Methods

**FIGURE 1:**
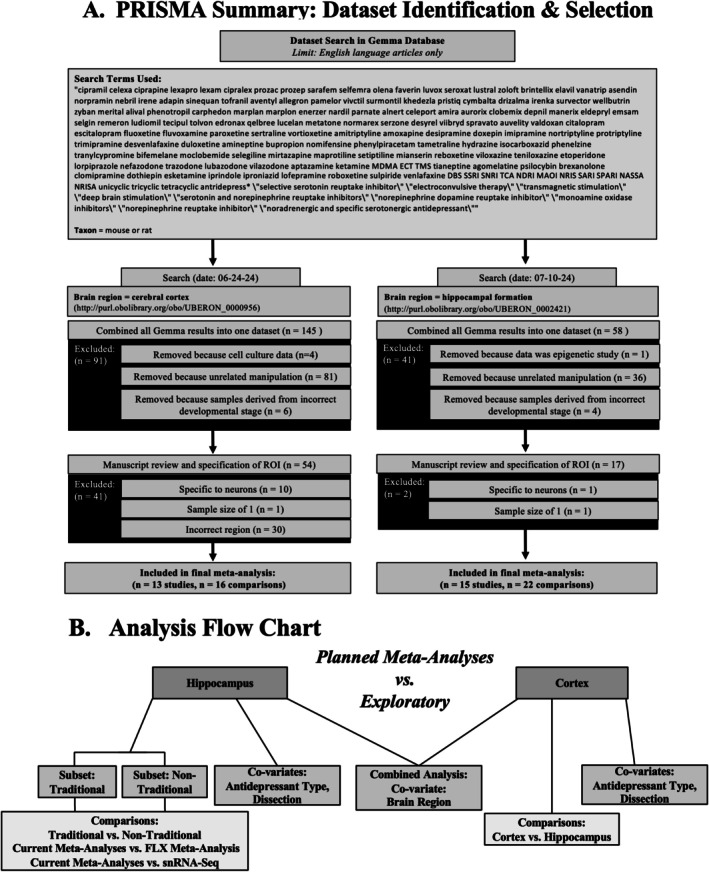
A PRISMA diagram overviewing hippocampal and cortical dataset search and selection. (A) We identified transcriptional profiling datasets of laboratory rodents treated with antidepressants within the Gemma database using pre‐specified keywords. The search encompassed tissue from the hippocampal formation. The titles, abstracts, and metadata for the datasets were initially scanned and filtered using pre‐specified inclusion/exclusion criteria, including indications that the dataset was an epigenetic study, had a manipulation unrelated to antidepressants, or had samples originating from the incorrect developmental stage or cell culture. The secondary screening step included a detailed review of the metadata on Gemma and published methodology, followed by a final specification of the region of interest (ROI: Hippocampus or cortex). Datasets were excluded in the secondary dataset filtering if they did not include a sufficient sample size, were cell type‐specific or sampled from a different region. (B) A flow chart overviewing the analyses, including planned meta‐analyses examining the converging effects of antidepressants on the hippocampus and cortex, and exploratory analyses comparing effects within particular antidepressant categories (traditional monoaminergic‐targeting vs. nontraditional mechanisms), dissections (dentate gyrus (DG) vs. hippocampus, anterior cingulate (ACG) vs. cortex, prefrontal cortex (PFC) vs. cortex), and brain regions (cortex vs. hippocampus). Exploratory analyses also compared meta‐analysis results to previous work, including a previous meta‐analysis examining the effects of FLX on the hippocampus (Ibrahim et al. 2022) and snRNA‐Seq studies examining the effect of FLX and ECT on hippocampal cell types (Nguyen, Sun, et al. 2025; Santiago et al. 2026). ACG, anterior cingulate cortex; DG, dentate gyrus; ECT, electroconvulsive therapy; FLX, fluoxetine; *n*, number of datasets; PFC, prefrontal cortex; ROI, region of interest.

The meta‐analysis project was conducted using a standardized pipeline for planned analyses (protocol: (Hagenauer, Manh Nguyen, et al. 2024), validation: (Rhoads et al. 2026), *not pre‐registered*). Additional exploratory analyses are denoted in Figure 1 and the text, with all analysis code (R v.4.4.1 and RStudio v.2024.9.9.365 (R Core Team 2024; RStudio Team 2024)) available at: https://github.com/evageoghegan/AntidepressantMetaAnalysis.

### 2.1 Information Source

The Gemma database was used to access curated and reprocessed public transcriptional profiling data (Lim et al. 2021; University of British Columbia 2024). Gemma contains differential expression results for nearly 20 000 publicly available transcriptional profiling datasets, representing approximately 600 000 samples (University of British Columbia 2024; Wickham et al. 2019). We focused on this database to provide consistency in our meta‐analysis input: Gemma adheres to a strict, standardized pipeline for dataset curation, preprocessing, and re‐analysis (Lim et al. 2021; University of British Columbia 2024). During preprocessing, Gemma realigns the datasets to an updated genome. Quality control measures include identification and removal of outlier samples, removal of genes (rows) with minimal variance in expression values (either zero variance or < 70% distinct values), and manual curation for common issues such as batch effects (Lim et al. 2021). Differential expression is calculated using the *limma* or *limma‐voom* pipeline, and statistical output is available for the full model (omnibus) and individual contrasts.

### 2.2 Dataset Identification and Search

For our meta‐analysis, we focused on treatments that specifically target depressive mood symptoms (Bains and Abdijadid 2023). A list of common traditional antidepressants, corresponding brand names, and drug classes was compiled (Figure 1). Nontraditional antidepressant treatments were added if there was approved clinical use or high‐quality peer‐reviewed clinical evidence available for potential antidepressant effects. These included ECT, TMS, DBS, ketamine, 3,4‐methylenedioxymethamphetamine (MDMA), psilocybin, tianeptine, quetiapine, and agomelatine (Guardiola‐Lemaitre et al. 2014; Gill et al. 2020; Tran and Argáez 2020; Kisely et al. 2018; Sonmez et al. 2019; Gassaway et al. 2014; Bahji et al. 2021; Pagnin et al. 2008; Patel and Titheradge 2015). The scope for the search included transcriptional profiling datasets from laboratory rats ( *Rattus norvegicus* ) or laboratory mice ( *Mus musculus* ) using bulk tissue dissections that targeted the full or large representative portion of the transcriptome. Search terms were initially generated by three of the authors (EMG, EH, and SE) and decisions reviewed and approved by co‐authors (MHH, RH, SE, MRB, SM). The search terms were then structured with Boolean operators (PRISMA diagram: Figure 1) and the search conducted using Gemma's application programming interface (API, packages *gemma.R* (v.3.1.6), *plyr* (v.1.8.9), and *dplyr* (v.1.8.9) (University of British Columbia 2024; Wickham et al. 2023; Wickham 2023)).

### 2.3 Initial Filtering

The metadata for the identified datasets was filtered by “organism part” using the formal hierarchical ontology term for the hippocampus (UBERON_0002421) or cerebral cortex (UBERON_0000956). Metadata records were then filtered down to publicly available datasets that were not labeled as problematic within the Gemma database. The identified datasets were scrutinized for inclusion/exclusion based on systematic criteria (Hagenauer, Manh Nguyen, et al. 2024). Initial filtering for the hippocampal meta‐analysis was performed by the first author (EMG), and then decisions were reviewed and approved by co‐authors (MHH, RH). Forty‐one studies were removed because either (1) treatment was administered during development, (2) brain collection was during development, (3) the focus was epigenetic, or (4) the manipulation was unrelated to antidepressant treatment. Initial filtering for the cortical meta‐analysis was performed by two researchers (SE, EMG), and then decisions were reviewed and approved by co‐authors (M.H.H., R.H., M.R.B.). Ninety‐one studies were removed because either (1) the manipulation was unrelated to antidepressant treatment, (2) brain collection was during development, or (3) the studies were in cell culture.

### 2.4 Secondary Filtering

For the remaining studies, metadata for the differential expression results were downloaded and reviewed to ensure that there was an experimental manipulation relevant to antidepressant treatment and related statistical contrasts. The publications associated with the datasets were also referenced, focusing exclusively on methodological information to determine inclusion/exclusion. Following this review, two datasets were removed from the hippocampal meta‐analysis: one due to being neuron‐specific and another due to having a sample size of *n* = 1 per subgroup. For the cortical meta‐analysis, 41 datasets were removed: 10 due to being neuron‐specific, 30 due to focus on noncortical regions, and one due to having a sample size of *n* = 1 per subgroup. At this stage, all inclusion/exclusion decisions were reviewed and finalized by the entire 2024 cohort of the *Brain Data Alchemy Project* (M.H.H., S.E., E.I.F., S.M., R.B., L.T.C., A.L., D.M.N., T.Q.D.) and laboratory principal investigator (R.H.).

### 2.5 Reprocessing

Four studies required reprocessing: GSE118670, GSE26836, GSE84183, and GSE43261. For GSE118670, GSE26836, and GSE43261, the differential expression model used by Gemma included multiple tissue types, and re‐analysis was necessary to extract tissue‐specific differential expression results. For GSE43261, the differential expression model also used treatment sensitivity phenotype as the variable of interest rather than the treatment itself. For GSE84183, the expression data appeared to have been log (2) transformed twice during Gemma's preprocessing, showing a highly reduced range.

To re‐analyze these datasets, we followed Gemma's analysis pipeline as much as possible to maintain consistency. We used the *Gemma.R* (v.0.99.41) and *tidyverse* (v.2.0.0) (Lim et al. 2021; Wickham et al. 2019) packages to import preprocessed log(2) expression data and sample metadata from the Gemma database (import date: October 22, 2024). Each dataset was subsetted to only our brain regions of interest, and the Gemma Diagnostics tab was consulted to determine whether outlier samples or batch effects were previously identified (*none found*). For GSE118670, adult and developing comparison mice were also excluded, as they did not receive antidepressant or control treatment conditions.

Genes (rows) were excluded that lacked log (2) expression data or had insufficient variability within the antidepressant or control groups. The control group for the antidepressant treatment was defined as the treatment factor reference level (intercept). Any additional experimental variables present in the metadata were included as co‐variates (GSE84183: stress treatment, with a reference level of no stress, GSE118670, GSE43261, and GSE26836: no co‐variates). The differential expression model was fit using the *limma* pipeline (package: *limma* v.3.17) with correction for heteroskedasticity (mean–variance trend) and an empirical Bayes correction (Ritchie et al. 2015).

### 2.6 Result Extraction and Meta‐Analysis

Differential expression results for the antidepressant treatment vs. control statistical contrasts from each included study were extracted. The Log (2) Fold Change (Log2FC) was extracted for each gene from each study, and standard error (SE) and sampling variance were calculated. If more than one result represented a gene, the Log2FC and SE were averaged. Results were then aligned across species using EntrezID and a version of the Jackson Labs Mouse Ortholog Database (Baldarelli et al. 2024) that had been trimmed to one‐to‐one orthologs (*Meta‐Analysis Input*: Table S1). For a gene to be included in the hippocampal or cortical meta‐analysis, a minimum number of 11 results (contrasts) needed to be available. A simple (intercept‐only) random effects meta‐analysis was then fit to the Log2FC estimates from each contrast (package: *metafor* (Viechtbauer 2010) (v.4.8.0)). A false discovery rate (FDR) correction using the Benjamin‐Hochberg method was applied to the nominal meta‐analysis (two‐tailed) *p*‐values (*multtest* v.1.32.0) (Pollard et al. 2005). Differentially expressed genes (DEGs) were defined using a threshold of FDR < 0.05, and visualized using forest plots (package: *metafor* (v.4.8.0) (Viechtbauer 2010)).

### 2.7 Assessment of Meta‐Analysis Result Robustness and Validity

To evaluate the robustness and validity of the meta‐analysis results, we examined the potential impact of publication bias and influential outlier studies. Publication bias was assessed using Egger's regression analysis (function *regtest()* in *metafor* (Viechtbauer 2010) v.4.8.0), which detects funnel plot asymmetry (Egger et al. 1997). Nominal Egger test (two‐tailed) *p*‐values were corrected for FDR using the Benjamin‐Hochberg method (*multtest* v.1.32.0) (Pollard et al. 2005). Genes with Egger FDR < 0.05 were considered to show evidence of potential publication bias. To assess the influence of individual studies on meta‐analysis results, the meta‐analysis was repeated while iteratively excluding each of the study contrasts (*leave1out()* function in *metafor* v.4.8.0 (Viechtbauer 2010)). If a previously‐identified DEG was found to drop below a nominal significance threshold (*p* > 0.05) following the exclusion of any of the study contrasts, the finding was considered to not be robust. To determine which of the study contrasts were disproportionately influential on the meta‐analysis results, we outputted Cook's Distances (Cook 1977) (Cook's D) and Difference in Betas (DFBetas), as well as a general assessment of overall influence (TRUE/FALSE) as provided by the *influence()* function (*metafor* v.4.8.0 (Viechtbauer 2010)).

### 2.8 Assessment or Residual Heterogeneity

To assess residual heterogeneity, we extracted statistics from the meta‐analysis object (*metafor* v.4.8.0 (Viechtbauer 2010)) estimating the residual variance in the underlying true antidepressant effect sizes (Log2FCs) observed across study contrasts, either in absolute terms (Tau^2^ and associated SE) or as a percent (*I* ^2^) or ratio (*H* ^2^) of the total variability. To determine whether this residual heterogeneity exceeded what might be expected due to random sampling variability, we extracted the Cochran's *Q* test statistic and associated nominal (two‐tailed) *p*‐value (Cochran 1954), which was corrected for FDR using the Benjamin‐Hochberg method (*multtest* v.1.32.0) (Pollard et al. 2005). Genes with a *Q*‐test FDR < 0.05 were considered to show significant heterogeneity across studies.

### 2.9 Exploratory Follow‐Up Analyses

In order to explore the effects of different treatment categories on the hippocampus, two subgroup meta‐analyses were run using only the contrasts characterizing the effects of: (1) traditional, monoaminergic‐targeting antidepressants, (2) nontraditional antidepressants (Table 1). Based on dataset availability, for a gene to be included in the traditional subgroup meta‐analysis, differential expression results needed to be available from a minimum of 12 antidepressant vs. control comparisons, whereas for the nontraditional subgroup meta‐analysis the minimum was 7 antidepressant vs. control comparisons. The effects of traditional versus nontraditional antidepressants were further compared using a hippocampal meta‐regression that included antidepressant type (nontraditional vs. traditional) and dissection (dentate gyrus vs. hippocampus) as co‐variates, with the general antidepressant effect (intercept) centered between the levels for each variable (i.e., at the average of the cell means: *contrast. sum*). This meta‐regression was designed to control for the correlation between antidepressant type and dissection method, as a larger percent (6 of 8: 75%) of the statistical contrasts for the nontraditional antidepressants used whole hippocampal tissue than for the traditional antidepressants (6 of 13: 46%). Two additional exploratory meta‐regressions were run that included the variables of platform (microarray vs. RNA‐seq) and depression model inclusion in the study (versus control‐only studies), but both variables were found to have minimal impact in our design (see the Supporting Information).

**TABLE 1:** Overview of datasets included in the meta‐analyses.

Dataset ID	Total *n*	HPC *n*	CTX *n*	Species	Sex	Authors	Year	Platform	Tissue	Model of stress/depression	Treatment
GSE109445	12	12 (9)	NA	Rat	M	Wang et al.	2018	Illumina (RNA‐Seq)	Ammon's horn	CUMS, Ctrl	**FLX (3), Ctrl (6)**
GSE123027	24	24	NA	Mouse	M	Rimmerman et al.	2021	Illumina (RNA‐Seq)	Ammon's horn	CUMS	*ECT* (10), *Ctrl* (14)
GSE205325	21	21	NA	Rat	M	Demin et al.	2022	Illumina (RNA‐Seq)	Ammon's horn	CUMS, CUMS‐LPS, Ctrl	**FLX (9), Ctrl (12)**
GSE230148	40	40	NA	Rat	M	Weiler et al.	2023	Agilent (microarray)	Ammon's horn	Aged w/ or w/o cog. impairment, Adult Ctrl	*TMS‐mod* (14), *TMS‐1 Hz* (12), *Ctrl* (14)
GSE230149	56	28	28	Rat	M	Weiler et al.	2023	Agilent (microarray)	Ammon's horn, Parietal cortex	Aged w/ or w/o without cog. impairment, Adult Ctrl	HPC: *TMS* (13), *Ctrl* (15)
											CTX: TMS (14), Ctrl (14)
GSE27532	16	16	NA	Mouse	M	Lisowski et al.	2011	Illumina (microarray)	DG	Bred for High/Low Stress Analgesia	**Desipramine (8), Ctrl (8)**
GSE56028	21	21	NA	Rat	M	Patrício et al.	2015	Agilent (microarray)	DG	CUMS, Ctrl	**Imipramine (3), FLX (3)**
											*Agomelatine* (3)
											*Tianeptine* (3), ***Ctrl (9)***
GSE61301	8	8	NA	Rat	M	Husain et al.	2015	Agilent (microarray)	Ammon's horn	Ctrl	Imipramine+yohimbine (4), Ctrl (4)
GSE63469	6	6	NA	Mouse	M	Proft et al.	2019	Affymetrix (microarray)	Ammon's horn	Stress sensitive (DBA/2) mice	**Venlafaxine‐ 100 kg/d/kg (2), Venlafaxine‐ 30 kg/d/kg (2), Ctrl (2)**
GSE73798	60	60 (24)	NA	Mouse	M	Ficek et al.	2019	Illumina (microarray)	Ammon's horn	Ctrl	*Ketamine* (12),*Ctrl* (12)
GSE81672	99	24	25	Mouse	M	Bagot et al.	2018	Agilent (microarray)	Ammon's horn, PFC	CSDS, Ctrl	HPC: *Ketamine* (6), **Imipramine (7), *Ctrl (11)***
											CTX: Ketamine (6), Imipramine (7), Ctrl (12)
GSE43261	38	38	NA	Mouse	M	Samuels et al.	2014	Affymetrix (microarray)	DG	CORT	HPC‐ventral: **FLX (11)**
											**Ctrl (8)**
											HPC‐dorsal: **FLX (11), Ctrl (8)**
GSE84183	64	32	32	Mouse	M	Hervé et al.	2017	Agilent (microarray)	DG, ACG	CUMS, Ctrl	HPC: **FLX (16), Ctrl (16)**
											CTX: FLX (16), Ctrl (16)
GSE118670	44	16	28 (16)	Mouse	M	Hagihara et al.	2019	Affymetrix (microarray)	DG, PFC	Ctrl	HPC: **FLX (8), Ctrl (8)**
											CTX: FLX (8), Ctrl (8)
GSE26836	12	6	6	Mouse	M	Chadwick et al.	2015	Illumina (microarray)	Ammon's horn, Frontal cortex	Aged Alzheimer's genetic model	HPC: **Amitriptyline (3), Ctrl (3)**
											CTX: Amitriptyline (3), Ctrl (3)
GSE28644	60	NA	60	Mouse	M	Benton et al.	2012	Affymetrix (microarray)	Cerebral cortex	30 inbred strains	FLX (30), Ctrl (3)
GSE93041	9	NA	9 (6)	Mouse	M	Orozco‐Solis et al.	2018	Affymetrix (microarray)	ACG	Ctrl	Ketamine (3), Ctrl (3)
GSE150264	36	NA	36	Mouse	F	Rajkumar et al.	2020	Agilent (microarray)	ACG	Genetic depression model, Ctrl	Imipramine (19), Ctrl (17)
GSE168172	15	NA	15	Mouse	M	Funayama et al.	2022	Illumina (RNA‐Seq)	PFC	Stress sensitive (BALB) mice	Duloxetine (6)
											Sertraline (6), Ctrl (3)
GSE138802	6	NA	6	Mouse	M	Kokkinou et al.	2021	Illumina (RNA‐Seq)	PFC	Ctrl	Ketamine (3), Ctrl (3)
GSE129359	24	NA	24 (12)	Mouse	M	Lukić et al.	2019	Illumina (RNA‐Seq)	PFC	Ctrl	Duloxetine (6), Ctrl (6)
GSE45229	20	NA	10 (6)	Mouse	M	Kondo et al.	2013	Affymetrix (microarray)	Frontal cortex	Ctrl	Quetiapine‐10 mg/kg (2)
											Quetiapine‐100 mg/kg (2), Ctrl (2)
GSE253280	36	NA	18 (12)	Rat	M	Warner‐Schmidt, et al.	2024	Illumina (RNA‐Seq)	Frontal cortex	Ctrl	MDMA (6), Ctrl (6)

*Note:* Color refers to datasets included in specific meta‐analyses: Hippocampal (yellow), hippocampal and cortical (green), cortical (blue). Experiment ID refers to the Gene Expression Omnibus (GEO) accession number for each dataset. The Author column refers to the first author of the publication where available (Wang et al. 2021; Rimmerman et al. 2022; Demin et al. 2022; Weiler et al. 2023; Lisowski et al. 2013; Patrício et al. 2015; Husain et al. 2015; Ficek et al. 2016; Bagot et al. 2017; Samuels et al. 2014; Hervé et al. 2017; Hagihara et al. 2019; Chadwick et al. 2011; Benton et al. 2012; Orozco‐Solis et al. 2017; Rajkumar et al. 2020; Funayama et al. 2023; Kokkinou et al. 2021; Lukić et al. 2019; Kondo et al. 2013; Warner‐Schmidt et al. 2024). The Year column refers to the dataset release date listed on Gemma. All experimental details describe the samples used in the transcriptional profiling experiment (e.g., species, biological sex). The Total n indicates the number of tissue samples in the full dataset, whereas the HPC n and CTX n indicate the number of hippocampal and cortical samples included in the full (omnibus) statistical model for each brain region, respectively, with the sample size in parentheses indicating the number of samples represented in the antidepressant treatment‐related statistical contrasts (if different from the full model). The Platform column indicates the transcriptional profiling technology used. The Tissue column indicates the specific region/subregion sampled. Model of Stress/Depression lists relevant subject characteristics or chronic stress conditions included in the experiment (acute stress due to behavioral assays or routine procedures not noted). Treatment column lists each treatment group (with # of samples) included in the treatment vs. control statistical contrasts used in the meta‐analysis. Treatment categories included SSRIs (FLX, sertraline), TCAs (imipramine, desipramine, amitriptyline), SNRIs (venlafaxine, duloxetine), NMDA receptor antagonists (ketamine), Alpha‐2 adrenergic antagonists combined with TCAs (yohimbine and imipramine), atypical antidepressants (tianeptine), MASSAs (agomelatine), atypical antipsychotics (quetiapine), serotonin–norepinephrine–dopamine releasing agent (SNDRA) (MDMA), and neuromodulatory brain stimulation (ECT, TMS). Within all hippocampal datasets (yellow and green), bolded treatments indicate inclusion in the traditional‐only subanalysis and italicized treatments indicate inclusion in the nontraditional only subanalysis.

Abbreviations: ACG, Anterior Cingulate Cortex; CSDS, chronic social defeat stress; Ctrl, Control; CUMS, chronic unpredictable mild stress; DG, dentate gyrus; ECT, Electroconvulsive therapy; FLX, fluoxetine; HPC, hippocampus; LPS, lipopolysaccharide (LPS)‐induced depression‐like model; MASSAs, melatonin agonist and selective serotonin antagonist; MDMA, 3,4‐Methylenedioxymethamphetamine; NMDA, N‐Methyl‐D‐aspartate; OBX, olfactory bulbectomy; PFC, Prefrontal Cortex; SIA, stress‐induced analgesia; SNDRA, serotonin–norepinephrine–dopamine releasing agent; SNRI, serotonin‐norepinephrine reuptake inhibitor; SSRI, selective serotonin reuptake inhibitor; TCA, Tricyclic antidepressant; TMS, Transcranial magnetic stimulation.

To further explore differences in antidepressant effects due to brain region, the contrasts from both hippocampus and cortex were included in a larger meta‐regression model that included region (hippocampus vs. cortex) as a co‐variate, with the general antidepressant effect (intercept) centered at the average of the cell means for the two regions (*contrast. sum*). For the cortical data, an exploratory meta‐regression was also performed to examine the effects of antidepressant type (traditional vs. nontraditional) while controlling for dissection (either ACG vs. other cortex or PFC vs. other cortex), but the small number of available contrasts suggested that we were underpowered to properly address this question (see the Supporting Information).

To compare the pattern of antidepressant differential expression identified in the different meta‐analyses (e.g., planned hippocampal and cortical meta‐analyses, exploratory traditional and nontraditional antidepressant meta‐analyses), Pearson's product moment correlation was calculated and visualized using scatterplots, and ranked gene lists compared using Spearman's rank correlation. To explore other potential sources of heterogeneity, hierarchically clustered heatmaps were created using the Log2FCs from each of the included studies for either all genes included in the hippocampal and cortical meta‐analyses or the following ranked gene lists: planned hippocampal meta‐analysis (all 58 significant DEGs), planned cortical meta‐analysis and exploratory subgroup meta‐analyses (top 50 genes, as ranked by *p*‐value).

### 2.10 Functional Patterns

Gene set enrichment analysis was used to determine whether the hippocampal and cortical meta‐analysis results, ranked by effect size (Log2FC), were enriched with down‐regulation or upregulation in sets of genes associated with known biological pathways and previous experimental results. Both directional (Log2FC) and nondirectional (abs(Log2FC)) versions of the analysis were carried out using the *fgsea* (v.1.32.0) package (Korotkevich et al. 2016) (parameters: nperm = 10 000, min size = 10, max size = 1000) and a gene set database including both traditional gene ontology gene sets and custom brain‐related gene sets (*Brain.GMT*: (Hagenauer et al. 2024)). *p*‐values were corrected for FDR using the Benjamini‐Hochberg method.

To explore cell type specificity, gene set enrichment analysis was also used to compare the results of the hippocampal meta‐analyses (full, traditional and nontraditional antidepressant meta‐analyses) to previously documented effects of FLX on hippocampal cell types (Nguyen, Sun, et al. 2025) or electroconvulsive shock (ECS) on hippocampal neurons (Santiago et al. 2026) as measured by single nucleus RNA‐Seq (snRNA‐Seq). Gene sets were constructed representing all genes that were significantly upregulated or downregulated in response to FLX (5 days of treatment or 3 weeks of treatment) or 10 sessions of ECS (20 days of treatment) in each of the hippocampal cell type clusters (46 gene sets total). Two gene sets were also constructed representing the “developmental‐like state” that was shown to increase in response to FLX and ECS, as measured using the top 100 genes upregulated and down‐regulated in postnatal day 5 vs. postnatal day 132 DG granule cells (Hochgerner et al. 2018). Enrichment within this custom gene set database was calculated using fGSEA using the procedure described above, but allowing for larger gene sets (max size = 2500), followed by FDR correction.

### 2.11 Comparison With Previous FLX Meta‐Analysis (Ibrahim Et al. 2022)

The effects of the FLX on the hippocampus (Ibrahim et al. 2022) were recently characterized using meta‐analyses of transcriptional profiling results from either stressed (7 studies, *n* = 75) or stress‐naive rodents (7 studies, *n* = 56). Due to differing inclusion/exclusion criteria, only 66% of the samples included in their meta‐analyses were in our own meta‐analysis (*n* = 87 of 131), contributing 28% to our final sample size (*n* = 87 of 313). Therefore, their results can provide insight that is partially independent from our own. Their publication provided results for the top DEGs identified using two methods of integrating findings across studies (integration method and portrait method) and consensus scores indicating whether those DEGs were predominantly upregulated or downregulated for the stressed (tables 2 and 3 in Ibrahim et al. 2022) and stress‐naive animals (tables 6 and 7 in Ibrahim et al. 2022). For comparison with our results, consensus scores from these tables were converted to a categorical variable indicating “Up” or “Down” direction of effect, which was then tested as a predictor for effects within our meta‐analyses (Log2FCs) using standard bivariate linear regression.

## 3 Results

### 3.1 Hippocampus

#### 3.1.1 Characteristics of the Datasets Included in the Hippocampal Meta‐Analysis

Fifty‐eight datasets were identified using prespecified antidepressant search terms (Figure 1). Of these, 15 datasets survived inclusion/exclusion criteria, leaving a final collective sample size of *n* = 313 hippocampal samples from antidepressant‐treated and control subjects. These datasets included 22 antidepressant vs. control comparisons (*full extracted results*: Table S1), representing 11 types of treatments, including pharmacological treatments (SSRIs, TCAs, SNRIs, NMDAR antagonists, Alpha‐2 adrenergic antagonists with TCAs, atypical antidepressants, and MASSAs) and nonpharmacological treatments (ECT, TMS) (Table 1), providing a diverse sample for examining the converging effects of different antidepressants on hippocampal gene expression.

The antidepressant treatments were tested using a variety of rodent depression models, including chronic unpredictable mild stress (CUMS), lipopolysaccharide‐induced depression‐like behavior, chronic corticosterone treatment, stress‐susceptible strains, and chronic social defeat stress (CSDS) (Rimmerman et al. 2022; Lisowski et al. 2013; Patrício et al. 2015; Bagot et al. 2017; Samuels et al. 2014; Hervé et al. 2017; Wang et al. 2022). Three datasets included aged subjects, some with transgenically‐induced cognitive impairment (Weiler et al. 2023; Chadwick et al. 2011). Three datasets utilized no depression model (Husain et al. 2015; Ficek et al. 2016; Hagihara et al. 2019). For species, most datasets used *Mus musculus* , with six using *Rattus norvegicus* . All samples were male (Table 1).

Other potential sources of heterogeneity in the datasets included platform and dissection. Most datasets were collected using microarray technology; the remaining three were by RNA‐seq. Two‐thirds were derived from whole Ammon's horn tissue, with the remaining specified as DG (Table 1).

#### 3.1.2 Meta‐Analysis Reveals Consistent Differential Expression Across Antidepressant Categories That Was Enriched in Many Cell Types and Pathways Previously Linked to Depression

**FIGURE 2:**
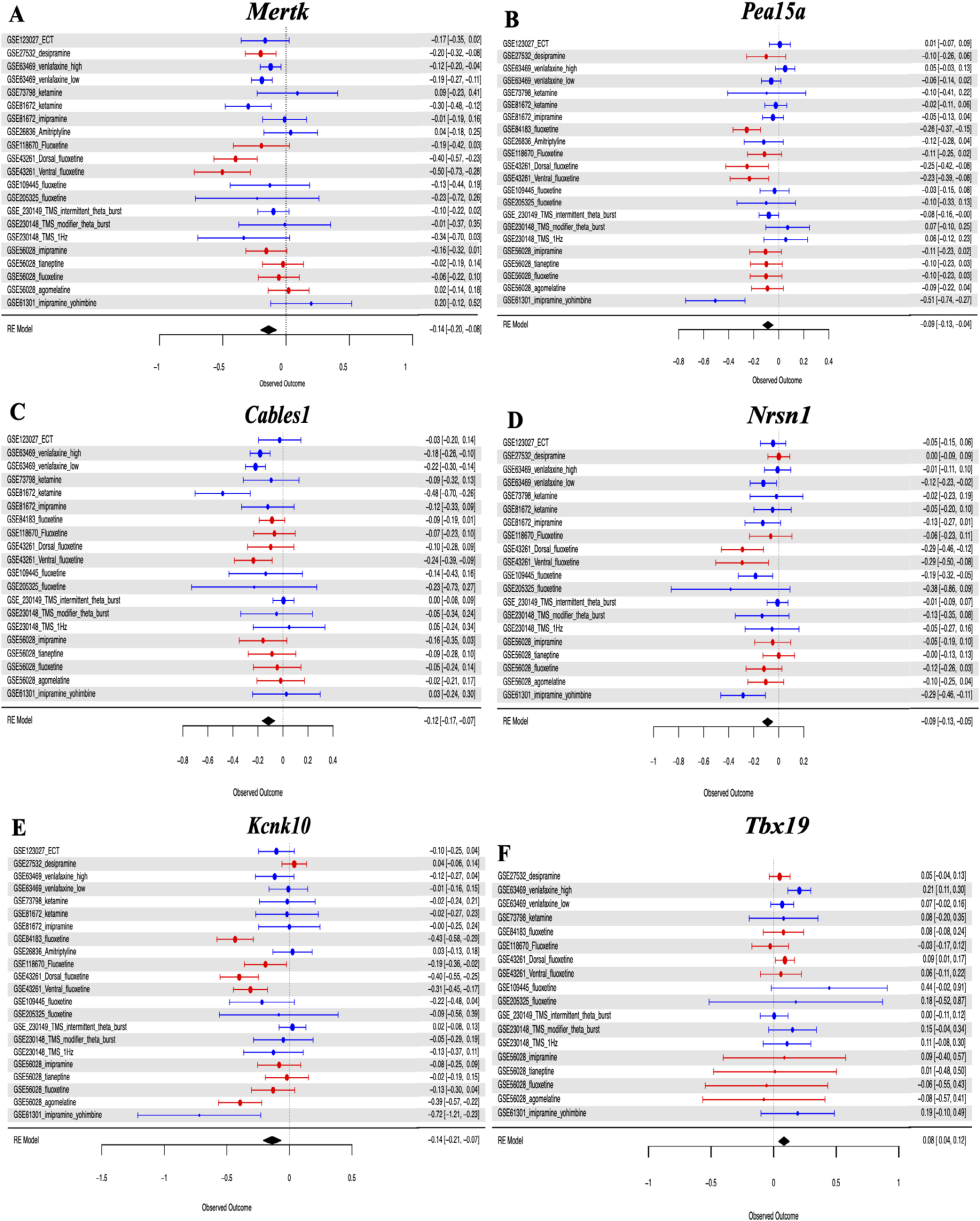
Hippocampal meta‐analysis reveals consistent differential expression across antidepressant categories: Example forest plots for six of the top DEGs (FDR < 0.05). Forest plots allow for visual inspection of the consistency and magnitude of antidepressant effects across all comparisons. Rows illustrate Log2FC (circles) with 95% confidence intervals (whiskers) for each of the antidepressant vs. control comparisons in each of the datasets and the meta‐analysis random effects model (“RE Model”). Color indicates tissue type, with DG in red and Ammon's horn in blue. The number of treatment vs. control comparisons that included measurements for the gene is determined following quality control. (A) *MER proto‐oncogene tyrosine kinase* (*Mertk*) was significantly downregulated across 21 comparisons. (B) *Proliferation and apoptosis adaptor protein 15A* (*Pea15a*) was significantly downregulated across 22 comparisons. (C) *Cdk5 And Abl Enzyme Substrate* (*Cables1*) was significantly downregulated across 20 comparisons. (D) *Neurensin 1* (*Nrsn1*) was significantly downregulated across 20 comparisons. (E) *Potassium Two Pore Domain Channel Subfamily K Member 10* (*Kcnk10*) was significantly downregulated across 22 comparisons. (F) *T‐box Transcription Factor 19* (*Tbx19*) was significantly upregulated across 18 comparisons. DEG, differentially expressed gene; DG, dentate gyrus; FDR, False Discovery Rate (*q*‐value); Log2FC, Log2 Fold Change (Antidepressant treatment vs. Control); RE Model, Random effects model.

**FIGURE 3:**
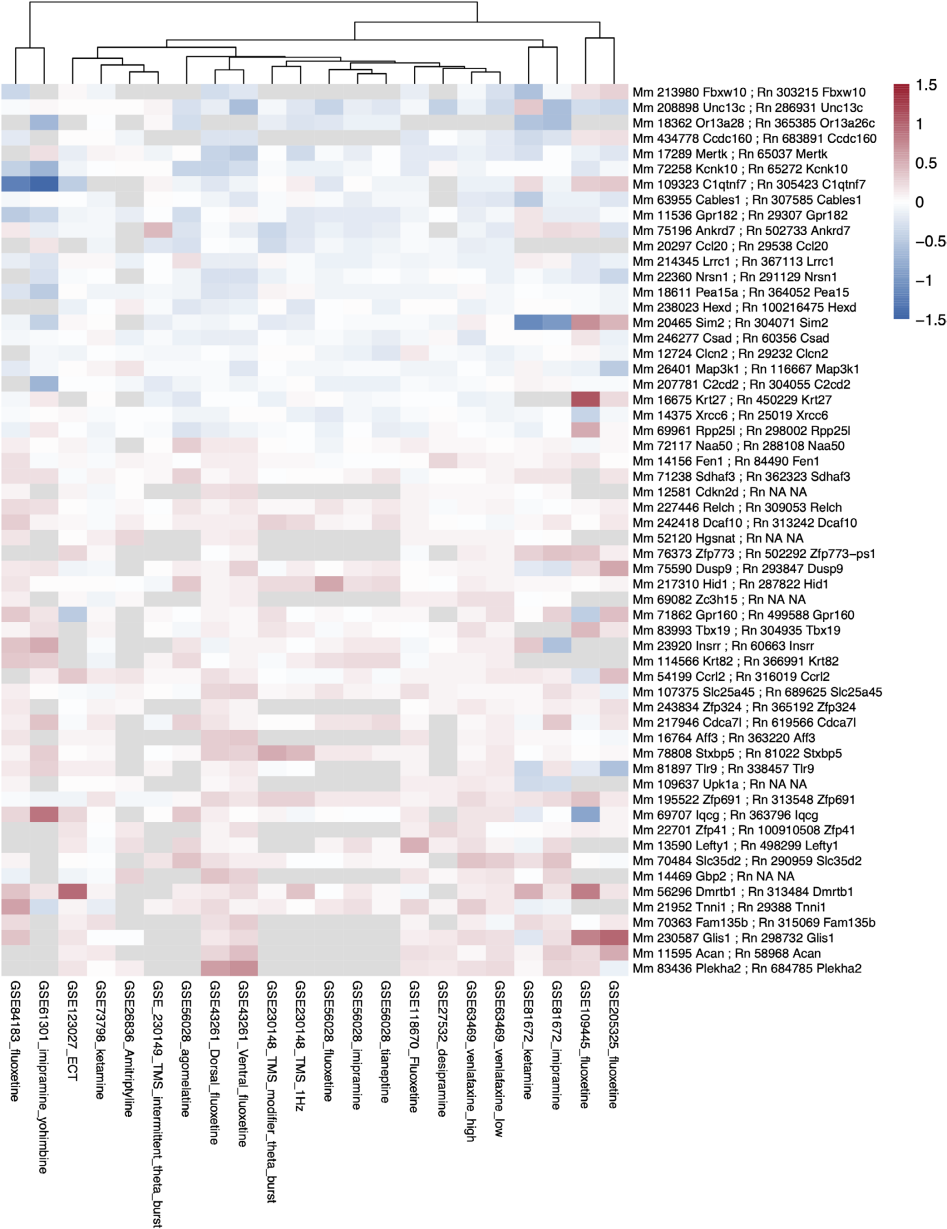
A hierarchically‐clustered heatmap of antidepressant effects across hippocampal datasets shows the consistency of effects across treatment type, tissue type, and platform for the top DEGs identified in the meta‐analysis. The heatmap allows for visual comparison of the expression patterns of the 58 hippocampal meta‐analysis DEGs (FDR < 0.05) across individual datasets. The color scale indicates standardized effect size (log_2_ fold change), with red denoting upregulation and blue denoting downregulation relative to control samples. Each column represents results from an individual antidepressant treatment (vs. control) comparison from the included datasets, as identified using the Gene Expression Omnibus accession # (GSE…). Each row represents one gene, identified by mouse (Mm) Entrez ID and official mouse gene symbol and rat (Rn) Entrez ID and official rat gene symbol, with NA values indicating a lack of clear ortholog in the species. At the top of the figure the dendrogram groups datasets by similarity in their transcriptional profiles, with shorter connecting lines indicating greater similarity in gene expression changes across datasets. DEG, differentially expressed gene; FDR, False Discovery Rate (*q*‐value); Mm, mouse; Rn, rat.

To be included in the meta‐analysis, a gene needed to be present in a minimum of 11 antidepressant vs. control comparisons. 16 494 genes fulfilled this requirement, and 16 439 genes produced stable meta‐analysis estimates (*full results*: Table S2). Fifty‐eight genes were significantly differentially expressed (FDR < 0.05, “DEGs”, Tables S3 and S4), of which 23 were consistently upregulated and 35 consistently downregulated following a variety of antidepressant treatments (*example forest plots*: Figure 2) often in 19 or more comparisons (*heatmap*: Figure 3).

This convergent antidepressant differential expression was enriched within 114 gene sets (nondirectional fGSEA analysis FDR < 0.05, *full results*: Table S5), many of which were associated with factors modulating depression risk. Of these enriched gene sets, 62 contained at least one significant antidepressant DEG amongst the “leading edge” genes driving the enrichment, and thus are of particular interest. The enriched gene sets containing DEGs spanned several categories (summarized in Table 2), including cell signaling (e.g., cytokine activity, calcium binding, enzyme‐linked kinase receptors), growth and development (e.g., cell proliferation and differentiation, morphogenesis, the DG), central nervous system cell types (e.g., pyramidal, GABA‐ergic, and dopaminergic neurons, astrocytes, myelinating cells, ependyma), and vasculature‐related cell types (e.g., endothelial cells, pericytes, fibroblasts). There was also enrichment in gene sets derived from previous differential expression studies, including several included in our meta‐analysis (2 gene sets, *not shown*), and studies of psychiatric disorders, depression, stress‐related animal models, and environmental enrichment. The directional version of the analysis identified 36 gene sets showing a nonsignificant trend towards enrichment with antidepressant effects (FDR < 0.1, *full results*: Table S6), 23 of which overlapped the nondirectional results (Table 2). These pathways enriched with convergent antidepressant differential expression are worth investigating further as potential linchpins for antidepressant efficacy.

**TABLE 2:**
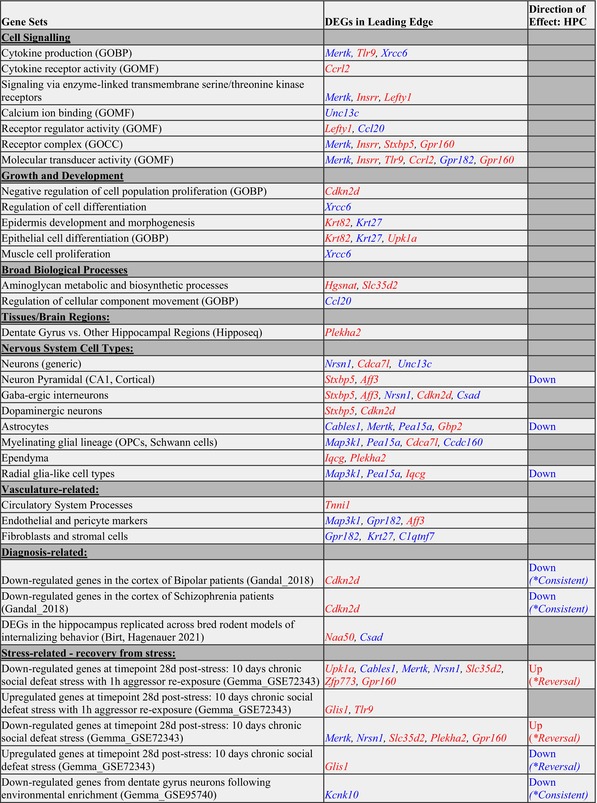
Convergent antidepressant effects on the hippocampus are enriched in many cell types and pathways, some of which have been previously linked to depression.

*Note:* Gene sets were identified as significantly enriched with antidepressant‐related differential expression in the hippocampal meta‐analysis via nondirectional functional gene set enrichment analysis (fGSEA, FDR < 0.05), and narrowed down to gene sets containing significant hippocampal antidepressant DEGs (FDR < 0.05) amongst the leading genes driving the enrichment. The direction of effect for the gene set is provided if the directional hippocampal fGSEA analysis trended towards significance (FDR < 0.10). Gene sets have been organized into groupings based on function, and filtered to reflect brain‐relevant functions. Gene set names have been reformatted and simplified for easy viewing, removing highly redundant terms with similar leading genes. Color indicates direction of effect; red: upregulated, blue: downregulated. Full results can be found in Tables S5 and S6.

Abbreviations: CA1, Cornu Ammonis 1; DEG, differentially expressed gene; FDR, False Discovery Rate (*q*‐value); fGSEA, fast gene set enrichment analysis; GOBP, Gene Ontology biological process; GOCC, Gene Ontology cellular component; GOMF, Gene Ontology molecular function; HPC, Hippocampus; OPC, Oligodendrocyte progenitor cell.

#### 3.1.3 Assessment of Result Robustness and Validity

Overall, the risk of systematic bias within the datasets from the individual transcriptional profiling studies was deemed low for any particular gene‐level result, as the original publications conducted full genome analyses and the data was re‐analyzed using a standardized pipeline. That said, the bias in the literature against negative results may inflate effect sizes represented in publicly‐released datasets, but in a manner that should be impartial to direction‐of‐effect. To double‐check our assumptions, we conducted an Egger's regression analysis to identify genes that might show evidence of funnel plot asymmetry, a commonly used metric indicating potential publication bias. We found 58 genes that showed evidence of publication bias (Egger's test FDR < 0.05: Table S7), several of which were immediate early genes (*Jun*, *Egr4*) or genes involved in neurotransmission (*Tdo2*, *Grm7*, *Calb1*, *Npy*). This could suggest that antidepressant datasets were more likely to be published or released if they supported neurotransmission‐related hypotheses. However, none of the genes with evidence of publication bias were among our antidepressant meta‐analysis DEGs (FDR < 0.05, *full results*: Table S2).

To assess the influence of individual studies and contrasts on meta‐analysis results, we repeated the meta‐analysis while iteratively excluding each of the study contrasts. We found that none of our meta‐analysis DEGs dropped below nominal significance (*p* < 0.05) following the removal of single study contrasts (maximum *p* = 0.0091), suggesting that our results were robust (*full results*: Table S2). We did not find evidence that small sample size studies were adding disproportionate noise in our meta‐analyses (Figures S1 and S2).

#### 3.1.4 Assessment of Sources of Antidepressant Effect Heterogeneity

Our meta‐analysis identified converging antidepressant effects across studies and treatments, but there was still evidence of residual heterogeneity. For example, although a hierarchically‐clustered heatmap of the top 58 antidepressant DEGs in our meta‐analysis did not show any obvious pattern in treatment type, platform, or tissue type (Figure 3), 14 of the DEGs still showed indications of significant true variation in antidepressant effects (Log2FC) across studies and contrasts as indicated by Cochran's *Q*‐test (FDR < 0.05). This variation was greater when considering the full 16 494 genes included in the meta‐ analysis, which had a median *I* ^2^ of > 50%, in a manner that was similarly not elucidated using simple hierarchical clustering (Figure S1). Therefore, we conducted a series of exploratory analyses to examine potential sources of effect heterogeneity.

#### 3.1.5 Exploratory Analyses: Traditional and Nontraditional Antidepressants Have Overlapping Effects on the Hippocampus

**FIGURE 4:**
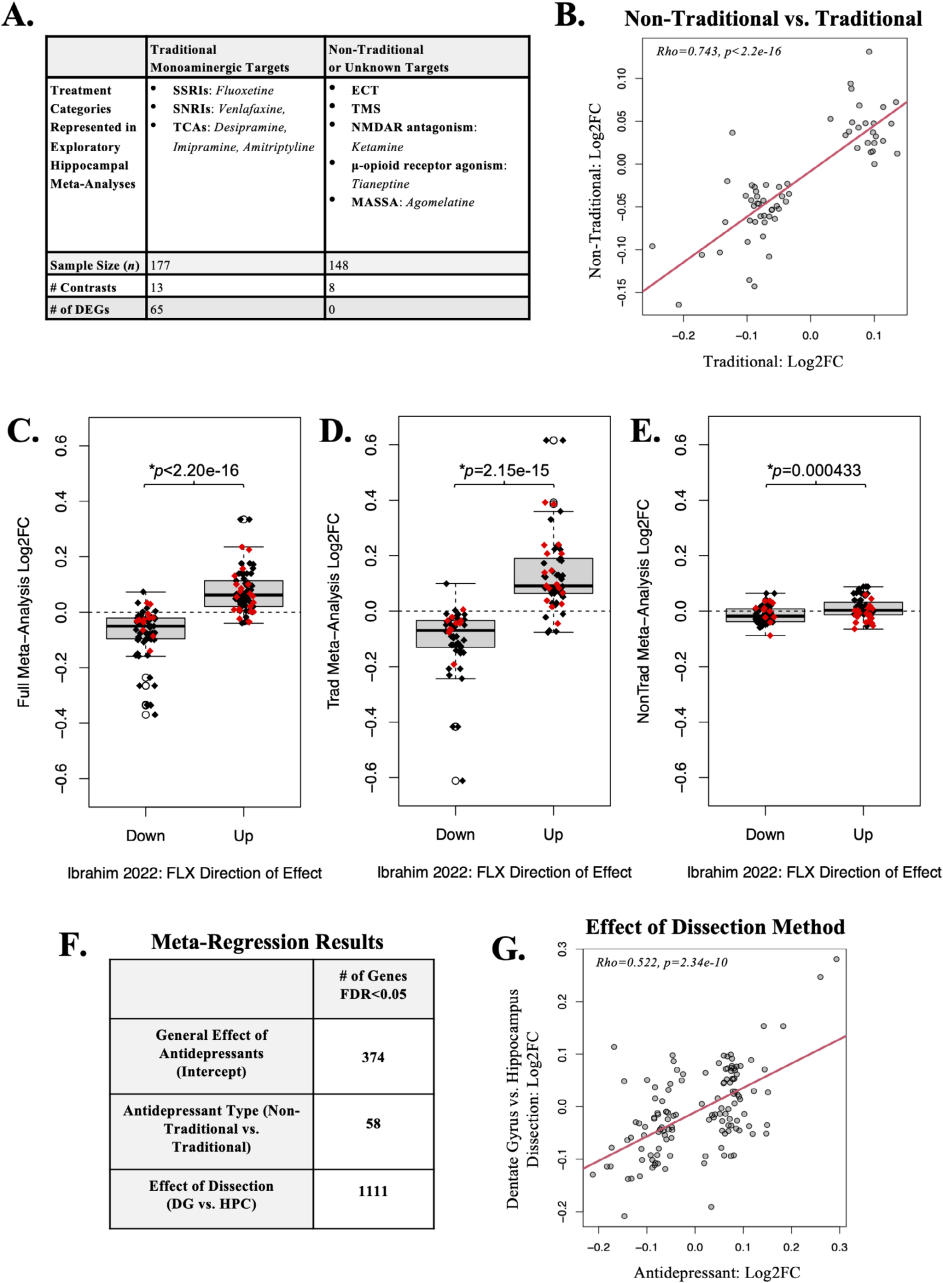
Exploratory analyses: Traditional and nontraditional antidepressants have overlapping effects on the hippocampus. (A) Table summarizing the types of antidepressant treatments, sample sizes, and number of antidepressant vs. control contrasts included for the exploratory meta‐analyses examining the effects of traditional (*n* = 177, 13 contrasts) and nontraditional (*n* = 148, 8 contrasts) antidepressants on the hippocampus. (B) Scatterplot comparing log_2_ fold changes from the traditional and nontraditional antidepressant treated hippocampus shows converging effects for the top antidepressant genes identified in our original meta‐analysis (FDR < 0.10: Rho = 0.743, *p* < 2.2e‐16). Convergence is also seen when comparing the effects of traditional and nontraditional antidepressants across all genes present in both analyses (Rho = 0.257, *p* < 2.2e‐16, Figure S5). (C–E) Boxplots with overlaid jittered data points illustrating that our meta‐analyses replicated the direction of effect observed within previous meta‐analyses of the effects of fluoxetine (FLX) on the hippocampus (Ibrahim et al. 2022). The previous meta‐analyses used a sample that only partially overlapped with our own (28%) and thus provide semi‐independent insight. The y‐axis shows the Log2FC estimated within our antidepressant meta‐analyses (C: full meta‐analysis, D: traditional antidepressant meta‐analysis, E: nontraditional antidepressant meta‐analysis) for each of the top genes identified within the previous FLX meta‐analyses of transcriptional profiling results from stressed (red) and stress‐naive (black) samples using both reported methods (“portrait” or “integration”). The *x*‐axis indicates the direction of effect identified by the consensus score in the FLX meta‐analyses. Boxes = first quartile, median, and third quartile; whiskers = range or 1.5× the interquartile range; open dot = outlier datapoint falling beyond the whiskers of the boxplot. (F) An exploratory meta‐regression controlling for heterogeneity introduced by antidepressant type (nontraditional vs. traditional) and dissection (dentate gyrus (DG) vs. whole hippocampus (HPC)) revealed more genes showing general antidepressant effects (FDR < 0.05) than our original hippocampal meta‐analysis, as well as many genes showing effects modulated by antidepressant type (FDR < 0.10) and dissection (FDR < 0.10). (G) The modulating effect of dissection (Log2FC) identified by the meta‐regression correlated with the overall effect of antidepressants (Log2FC) both for the top genes identified in our original meta‐analysis (scatterplot: Genes with original FDR < 0.10: Rho = 0.522, *p* = 2.34e‐10) and for all genes in the analysis (Figure S8: Rho = 0.498, *p* < 2.2e‐16) in a manner suggesting that antidepressant effects were larger in DG dissections than in the whole hippocampus. DEG, differentially expressed gene; DG, dentate gyrus; ECT, Electroconvulsive therapy; FDR, False Discovery Rate (*q*‐value); FLX, Fluoxetine; HPC, hippocampus; Log2FC, Log (2) Fold Change; MASSAs, melatonin agonist and selective serotonin antagonist; *n*, sample size; NMDA, N‐Methyl‐D‐aspartate; NonTrad, nontraditional antidepressants; *p*, nominal *p*‐value; Rho, Spearman rank correlation; SNRI, serotonin‐norepinephrine reuptake inhibitor; SSRI, selective serotonin reuptake inhibitor; TCA, Tricyclic Antidepressant; TMS, Transcranial magnetic stimulation; Trad, Traditional mono‐aminergic targeting antidepressants.

As an exploratory follow‐up analysis, we compared the patterns of gene expression associated with traditional, mono‐aminergic targeting antidepressants and nontraditional antidepressants, both pharmacological and neuromodulatory (Table 1, Figure 4A). In the traditional antidepressant meta‐analysis, 10 datasets were included, with a collective sample size of *n* = 177 samples used in 13 antidepressant vs. control comparisons. In the nontraditional antidepressant meta‐analysis, six datasets were included, with a collective sample size of *n* = 148 samples used in eight antidepressant vs. control comparisons. For a gene to be included in the traditional subgroup meta‐analysis, differential expression results needed to be available from a minimum of 12 antidepressant vs. control comparisons. Of the 9628 genes fulfilling this requirement, 9612 produced stable meta‐analysis estimates. Sixty‐five of these genes were significantly differentially expressed (FDR < 0.05: 42 upregulated, 23 downregulated: Figure S3, *full results*: Table S8). For the nontraditional subgroup meta‐analysis, a gene needed to be present in a minimum of 7 antidepressant vs. control comparisons. Of the 12 203 genes fulfilling this requirement, 12 154 produced stable meta‐analysis estimates. None of these genes were significantly differentially expressed (Figure S4, *full results*: Table S9), but the pattern of nontraditional antidepressant‐related expression moderately resembled that observed in the traditional subgroup meta‐analysis when considering all genes present in both analyses (df = 12 614, *Rho* = 0.257, *p* < 2.2e‐16, Figure S5). As would be expected, both traditional and nontraditional antidepressants showed converging antidepressant effects for the top DEGs identified in our original meta‐analysis (e.g., FDR < 0.10 in original analysis: df = 61, *Rho* = 0.743, *p* < 2.2e‐16: Figure 4B).

A meta‐regression that included antidepressant type (nontraditional vs. traditional) and dissection (DG vs. whole hippocampus) as co‐variates showed even stronger findings (Figure S6). The general antidepressant effects detected within the meta‐regression with co‐variates were very similar to the antidepressant effects detected in our original meta‐analysis (Figure S7A: df = 16 401, *Rho* = 0.900, *p* < 2.2e‐16), and the predicted effects of traditional and nontraditional antidepressants paralleled our previous subsetted meta‐analysis findings (Figure S7B: traditional: df = 9594, *Rho* = 0.954, *p* < 2.2e‐16, Figure S7C: nontraditional: df = 12 123, *Rho* = 0.779, *p* < 2.2e‐16). However, more genes now showed a significant overall effect of antidepressants (374 genes *FDR* < 0.05) due to better controlling for heterogeneity, with 58 genes showed a modulating effect of antidepressant type (*FDR* < 0.05) and 1111 genes showed a modulating effect of dissection (*FDR* < 0.05, Figure 4F, *full results*: Table S10). Notably, the modulating effect of dissection correlated with both our original antidepressant meta‐analysis estimates (Figure S8A: all genes: df = 16 401, *Rho* = 0.362, *p* < 2.2e‐16, original top DEGs (*FDR* < 0.10): df = 128, *Rho* = 0.343, *p* = 6.98e‐05) and current antidepressant meta‐regression estimates (Figure S8B: all genes: df = 16 440, *Rho* = 0.498, *p* < 2.2e‐16, original top DEGs (*FDR* < 0.10): df = 128, *Rho* = 0.522, *p* = 2.34e‐10, Figure 4G) in a manner suggesting that antidepressant effects were larger in DG dissections than in the whole hippocampus.

We attempted exploratory meta‐regression analyses to examine other potential sources of effect heterogeneity (platform, inclusion of a depression model) but found little evidence of impact, most likely because these variables were not well represented in our design (Figure S9, *full results*: Tables S11 and S12).

#### 3.1.6 Exploratory Analysis: The Pattern of Effects Detected in Previous FLX Meta‐Analyses Appear Partially Generalizable to Other Antidepressants

The effects of FLX on hippocampal gene expression in stressed and stress‐naive animals were recently characterized using meta‐analyses of public transcriptional profiling studies (Ibrahim et al. 2022). When comparing our meta‐analysis findings to their results, we found reasonable convergence, despite only partially overlapping samples (28%). Overall, we tended to observe the same direction of effect (Table S13), with more positive Log2FCs observed in our meta‐analyses for genes previously found to be upregulated with FLX vs. down‐regulated with FLX (Figure 4C, *full meta‐analysis*: *R* ^2^ = 0.438, *β* ± SE = 0.149 ± 0.0146, *T*(135) = 10.25, *p* < 2.2e‐16). This similarity was stronger with the results of our meta‐analysis using only traditional antidepressants (Figure 4D, *R* ^2^ = 0.447, *β* ± SE = 0.237 ± 0.0254, *T*(105) = 9.311, *p* < 2.15e‐15), which had an overlapping sample, but still present—although weaker—in our meta‐analysis using nontraditional antidepressants (Figure 4E, *R* ^2^ = 0.0969, *β* ± SE = 0.0227 ± 0.00627, *T*(122) = 3.618, *p* = 0.000433). There was no overlap with our top DEGs, but 22 of the FLX genes showed the same direction of effect and nominal significance (*p* < 0.05) in our full hippocampal meta‐analysis, and only one showed the opposite direction of effect and nominal significance, providing suggestive support.

#### 3.1.7 Exploratory Analysis: Antidepressant Effects in the Current Hippocampal Meta‐Analyses Reproduce Effects Detected in Individual Cell Types Using snRNA‐Seq

**FIGURE 5:**
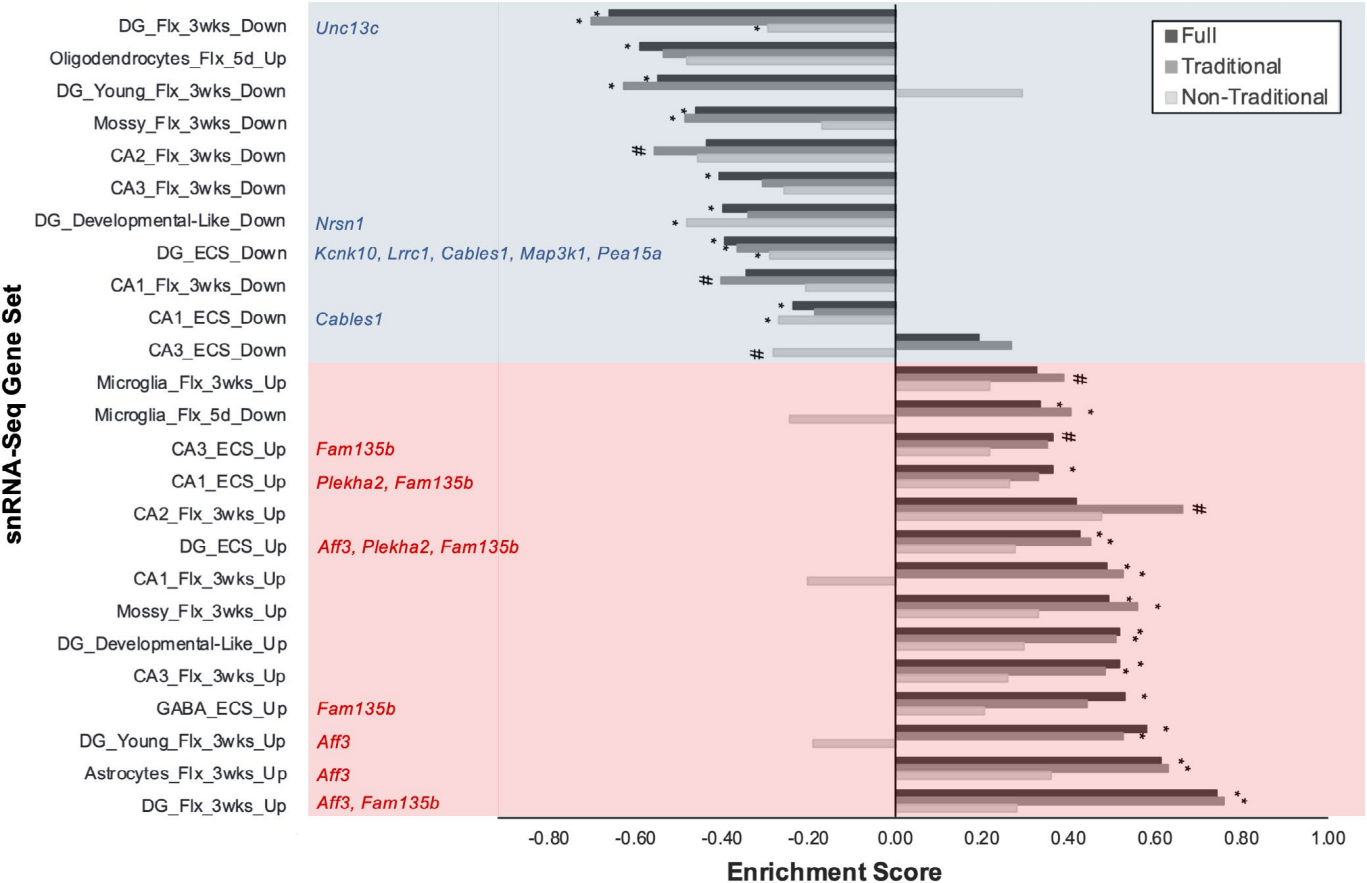
Exploratory analysis: Meta‐analyses of antidepressant effects identified in bulk hippocampal tissue uncover similar patterns of differential expression as those previously identified in specific hippocampal cell types using snRNA‐Seq. Sets of genes that were found to be differentially expressed in hippocampal cell types in response to fluoxetine (FLX: 5 days (5 d) or 3 weeks (3 wks) of treatment) or electroconvulsive shock (ECS, 10 days) as measured by snRNA‐Seq were enriched with similar differential expression in our hippocampal antidepressant meta‐analyses (full meta‐analysis, exploratory meta‐analysis focused on traditional antidepressants, exploratory meta‐analysis focused on nontraditional antidepressants). For ease of visualization, only gene sets with at least nominal enrichment (#*p* < 0.05) of differential expression in at least one of the meta‐analyses were included in the figure, with asterisks indicating enrichment surviving false discovery rate correction (*FDR < 0.05). In general, the direction of effect for the enrichment in the antidepressant meta‐analyses tended to mirror the direction of effect observed in the original snRNA‐Seq results. DG, Dentate Gyrus (granule cells); Mossy, Mossy Cells; CA, Cornu Ammonis (neurons); GABA, GABAergic interneurons; wks, weeks; d, days.

To follow up on the enrichment of antidepressant effects within gene sets associated with particular hippocampal cell types, we compared our meta‐analysis results to the effects of antidepressant treatments observed in two snRNA‐Seq studies: the effects of FLX (5 days or 3 weeks of treatment) on all hippocampal cell types (Nguyen, Sun, et al. 2025) and the effects of ECS (10 sessions over 20 days of treatment) on hippocampal neuronal cell types (Santiago et al. 2026). We found that differential expression within the antidepressant meta‐analyses was enriched within sets of genes that responded to 3 weeks of FLX and ECS treatment in multiple hippocampal cell type clusters (Table S14), including DG granule neurons, astrocytes, and other hippocampal neuron types (CA1, CA3, Mossy, GABA‐ergic), and within sets of genes responding to 5 days of FLX treatment in microglia and oligodendrocytes. Furthermore, the enrichment of upregulation or downregulation within the meta‐analyses results always paralleled the direction of effect observed in the snRNA‐Seq experiments for treatments with demonstrable antidepressant efficacy (3 weeks of FLX and ECS). There was also an enrichment of antidepressant effects within two gene sets previously identified as signifying the re‐activation of a “developmental‐like state” in DG granule neurons (Santiago et al. 2026; Nguyen, Sun, et al. 2025), which was theorized to mediate the antidepressant effects of FLX (Nguyen, Sun, et al. 2025) and ECS (Santiago et al. 2026) (Figure 5).

There was evidence of treatment heterogeneity: in general, the traditional antidepressant meta‐analysis results tended to more closely resemble the FLX snRNA‐Seq results than the nontraditional meta‐analysis results (Figure 5, Table S14). This might be expected due to FLX targeting traditional monoaminergic systems (SSRI). However, a similar bias was not observed for the ECS‐derived gene sets, and the full meta‐analysis results were strongly enriched with differential expression in gene sets derived from both FLX and ECS snRNA‐Seq experiments. Notably, genes that were down‐regulated in DG granule neurons following 3 weeks of FLX or ECS showed enrichment within all three of our hippocampal meta‐analyses (full, traditional, and nontraditional).

### 3.2 Cortex

#### 3.2.1 Characteristics of the Datasets Included in the Cortical Meta‐Analysis

Using prespecified antidepressant search terms, 147 datasets were identified (Figure 1). Of these datasets, 13 survived inclusion/exclusion criteria, leaving a final collective sample size of *n* = 233 cortical samples from antidepressant‐treated and control subjects. These 13 datasets included 16 antidepressant vs. control comparisons (*full extracted results*: Table S1) featuring a variety of antidepressants including pharmacological treatments (SSRIs, SNRIs, TCAs), atypical antipsychotics, NMDA receptor antagonists, serotonin–norepinephrine–dopamine releasing agent (SNDRA), and nonpharmacological treatments (TMS) (Table 1), providing a diverse sample for examining the converging effects of different antidepressants on cortical gene expression.

Unlike the hippocampal studies, most cortical studies (representing 10 of 16 contrasts) tested the effects of antidepressants without using depression models (Weiler et al. 2023; Hagihara et al. 2019; Chadwick et al. 2011; Benton et al. 2012; Orozco‐Solis et al. 2017; Rajkumar et al. 2020; Funayama et al. 2023; Kokkinou et al. 2021; Lukić et al. 2019; Kondo et al. 2013; Warner‐Schmidt et al. 2024), but sometimes used samples representing other characteristics including aged transgenic mice (Chadwick et al. 2011) and inbred strains (Benton et al. 2012). The remainder used CSDS and CUMS (Bagot et al. 2017; Hervé et al. 2017), a genetic depression model (*Brd1*+/− mice) (Rajkumar et al. 2020), and stress‐sensitive strain (Funayama et al. 2023). For species, all but two of the datasets used *Mus musculus* ; the remainder used *Rattus norvegicus*. With one exception, the datasets used only males (Table 1).

Other potential sources of heterogeneity in the datasets included platform and dissection. Most datasets were collected using microarray technology, with four using RNA‐Seq. Most datasets focused on the frontal cortex (11), but dissection varied considerably: four datasets focused on the PFC, one on the prelimbic subregion, and three on the anterior cingulate cortex (ACG). There was also a dataset from the parietal cortex, and “cerebral cortex,” broadly defined (Table 1).

#### 3.2.2 Cortical Meta‐Analysis Reveals One Gene Consistently Upregulated by Antidepressants (Atp6v1b2)

**FIGURE 6:**
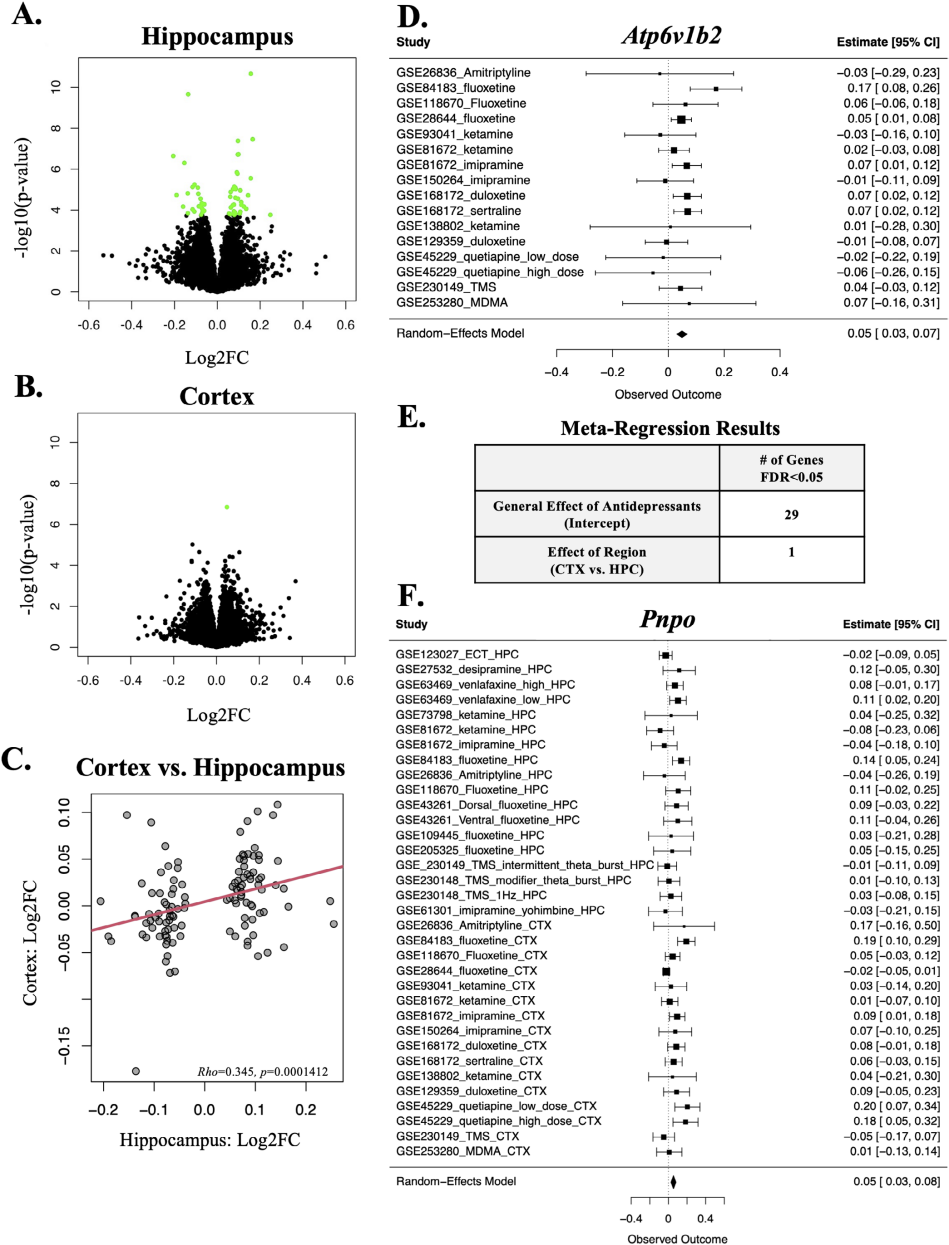
The cortex had fewer significant antidepressant effects, but showed some parallels with the hippocampus. (A, B) There were more antidepressant DEGs identified in the hippocampus than in the cortical meta‐analysis. Volcano plots display differential expression results for hippocampal (A) and cortical (B) meta‐analyses, with log_2_ fold change plotted against –log_10_(*p*‐value). Genes reaching significance at FDR < 0.05 are highlighted in green. (C) A scatterplot illustrates the positive correlation between the antidepressant effects (log_2_ fold changes or Log2FC) derived from the hippocampal and cortical meta‐analyses. This is true when considering either the top‐ranking antidepressant genes identified in the hippocampal meta‐analysis (depicted above: FDR < 0.10 in the original analysis: Rho = 0.345, *p* = 0.0001412) or all 14 436 genes included in both analyses (Rho = 0.292, *p* < 2e‐16, Figure S16). (D) Forest plot illustrating the antidepressant effects for *Atp6v1b2*, the one gene that reached significance in the cortical meta‐analysis (FDR < 0.05), which was upregulated across 16 comparisons. Rows illustrate Log2FC (circles) with 95% confidence intervals (whiskers) for each of the datasets and the meta‐analysis random effects model (“RE Model”). (E) A table showing the results from an exploratory meta‐regression using all 38 antidepressant vs. control contrasts derived from both the hippocampus and cortex, including brain region as a co‐variate. Out of the 12 857 genes included in the meta‐regression, 29 showed an overall effect of antidepressants and 1 showed a modulating effect of brain region. (F) A forest plot illustrating the small but consistent upregulation of *Pyridoxamine 5′‐phosphate oxidase* (*Pnpo*) following antidepressant treatment in datasets derived from both the hippocampus (HPC) and cortex (CTX) in the cross‐regional meta‐regression (FDR < 0.05). The enzyme encoded by *Pnpo* catalyzes the terminal, rate‐limiting step in the synthesis of vitamin B6. CTX, cortex; DEG, differentially expressed gene; ECT, Electroconvulsive therapy; FDR, False discovery rate (*q*‐value); HPC, hippocampus; Log2FC, Log(2) Fold Change; MDMA, 3,4‐methylenedioxymethamphetamine; *p*, nominal *p*‐value; RE Model, random‐effects model; Rho, Spearman rank correlation; TMS, transmagnetic stimulation.

To be included in the cortical meta‐analysis, a gene needed to be present in a minimum of 11 antidepressant vs. control comparisons. 15 583 genes fulfilled this requirement, and 15 454 genes produced stable meta‐analysis estimates (Figure 6B, *full results*: Table S15). Only one gene, *ATPase H+ transporting V1 subunit B2* (*Atp6v1b2*), was significantly differentially expressed (*FDR* < 0.05), showing upregulation across 16 comparisons (Figure 6D, *heatmap*: Figure S10). There were no gene sets that were significantly enriched with antidepressant differential expression in the directional or nondirectional fGSEA analysis (*FDR* < 0.05, *full results*: Tables S16 and S17).

#### 3.2.3 Assessment of Result Robustness and Validity

Similar to the hippocampus, the overall risk of systematic bias within the cortical datasets from the individual transcriptional profiling studies was deemed low for any particular gene‐level result, as the results were derived from full genome analyses. That said, an Egger's regression analysis identified seven genes with evidence of funnel plot asymmetry (FDR < 0.05: Table S18), providing weak evidence suggesting potential publication bias, but perhaps only in favor of datasets with overall larger effect sizes, as none of the seven genes had clear theoretical connections to antidepressant mechanisms. *Atp6v1b2* was not included amongst these genes (*full results*: Table S15).

To assess the influence of individual studies and contrasts on meta‐analysis results, we repeated the meta‐analysis while iteratively excluding each of the study contrasts. We found that *Atp6v1b2* did not drop below nominal significance (*p* < 0.05) following the removal of single study contrasts (maximum *p* = 0.000127), suggesting that the result is robust (*full results*: Table S15). We did not find evidence that small sample size studies were adding disproportionate noise in our meta‐analysis (Figures S11 and S12).

#### 3.2.4 Assessment of Sources of Antidepressant Effect Heterogeneity

The relative lack of converging antidepressant effects across cortical studies and treatments might be due to residual heterogeneity. However, the median I^2^ for the cortical meta‐analysis was less than what we observed in the hippocampus (38% vs. 50%, Figure S11), especially when considering the top antidepressant‐related genes as ranked by *p*‐value (e.g., the top 58 genes in the hippocampus meta‐analysis (~20%) vs. cortical meta‐analysis (0.02%)). Likewise, a hierarchically‐clustered heatmap of either the top antidepressant genes (Figure S10) or all 15 583 included in the cortical meta‐analysis (Figure S11) showed no obvious pattern related to treatment type, platform or tissue type. We attempted exploratory meta‐regression analyses to examine potential sources of effect heterogeneity (antidepressant type, dissection) akin to what was conducted for the hippocampus, but found little evidence of impact (antidepressant type, PFC dissection, Figure S13, *full results*: Table S19) or found potential impact but based on minimal observations (ACG dissection, Figures S14 and S15, *full results*: Table S20).

#### 3.2.5 Antidepressant Effects in the Cortex Show Some Resemblance to the Hippocampus

Despite the relative lack of significant antidepressant DEGs in the cortical meta‐analysis, there was a positive correlation between the hippocampal and cortical meta‐analysis results when considering either the top antidepressant genes identified in the hippocampal meta‐analysis (*FDR* < 0.10 in the original analysis: df = 116, *Rho* = 0.345, *p* = 0.0001412, Figure 6C) or all genes included in both analyses (df = 14 434, *Rho* = 0.290, *p* < 2e‐16, Figure S16A). To explore this similarity further, we conducted a meta‐regression using all 38 antidepressant vs. control contrasts derived from both the hippocampus and cortex, including brain region as a co‐variate. Out of the 12 857 genes included in the meta‐regression, 29 showed an overall effect of antidepressants (*FDR* < 0.05, Figure 6D, *full results*: Table S21), seven of which had been previously identified in our hippocampal meta‐analysis. In general, these antidepressant effect sizes tended to be small, with all but 3 of the DEGs showing an *abs*(Log2FC) < 0.10 (example forest plot: Figure 6E, other examples: Figure S16B–D). Only a single gene showed a significant modulating effect of brain region (*FDR* < 0.05) (*Sema4d*: *Semaphorin‐4D*), although 20 of the top antidepressant DEGs identified in the previous hippocampal meta‐analysis showed a nominal modulating effect of region in the meta‐regression (*p* < 0.05), suggesting effects were smaller in the cortex. Altogether, these findings imply that broadly‐defined brain region (hippocampus vs. cortex) is not a primary source of antidepressant effect heterogeneity in these bulk dissections, whereas hippocampal subregion (DG vs. hippocampus) appears more impactful.

## 4 Discussion

Our meta‐analyses provide a view into the shared mechanisms of action of diverse antidepressant treatments in the hippocampus and cortex. A wide variety of treatments were represented, including SSRIs, SNRIs, TCAs, NMDA receptor agonists, alpha‐2 adrenergic antagonists combined with TCAs, MASSAs, ECT, and TMS. This aggregation of data produced enhanced statistical power, with a collective sample size of *n* = 313 (hippocampus) and *n* = 233 (cortex). The hippocampal meta‐analysis identified 58 DEGs, many of which were associated with gene sets mediating factors known to modulate depression risk, such as stress, environmental enrichment, and other psychiatric disorders, as well as pathways previously linked to antidepressant efficacy, including DG granule neurons, cell proliferation, morphogenesis, vasculature, glia, synaptic plasticity, and immune signaling. Exploratory analyses further highlighted the importance of the DG in antidepressant effects. Our hippocampal meta‐analysis also confirmed patterns of gene expression identified in previous antidepressant studies, including a meta‐analysis of the effects of the FLX on the hippocampus (Ibrahim et al. 2022) and snRNA‐Seq studies characterizing the effects of FLX and ECS on hippocampal cell types (Nguyen, Sun, et al. 2025; Santiago et al. 2026), demonstrating that these effects can be generalized to a broad swath of antidepressants.

In the cortex, antidepressant effects resembled the hippocampus in some ways, but there was only one significant DEG, *Atp6v1b2*. When an exploratory meta‐regression was performed using both cortical and hippocampal datasets, there were many antidepressant effects that were consistent across both brain regions, with weak evidence of broad regional variation. Altogether, our results indicate that different antidepressant treatments converge on a variety of genes and pathways, with many effects shared across the hippocampus and cortex but varying at the level of subregion and cell type.

### 4.1 Hippocampus: Differential Expression Related to the Neurogenic Niche in the DG

Neuroimaging studies have consistently shown hippocampal volume reduction in patients with depression, with stress playing a key role (Belleau et al. 2019; Sahay and Hen 2007), and antidepressants producing a reversal (Sahay and Hen 2007; Sapolsky 2001; Czéh et al. 2001). Several theories of antidepressant mechanism center on the promotion of growth‐related processes and AHN (Sahay and Hen 2007; Santarelli et al. 2003). Therefore, it is interesting that multiple genes and cell types related to the neurogenic niche in the DG were highlighted in our meta‐analysis results, and our exploratory meta‐regression suggested that antidepressant effects were larger in DG dissections. Antidepressant treatment upregulated gene expression specific to the DG, including our most upregulated DEG (*Pleckstrin Homology Domain Containing A2 (Plekha2)*). Antidepressant treatment also altered expression specific to a variety of support cell types that play a critical role in the neurogenic niche, including vasculature‐related cells (endothelial cells, pericytes, astrocytes) that secrete factors that promote cell proliferation and differentiation (Ehret et al. 2015; Palmer et al. 2000; Chen et al. 2025) and regulate neural stem cell exposure to blood‐borne substances (Chen et al. 2025; Karakatsani et al. 2023; Licht and Keshet 2015), and radial glia‐like cells, which can serve as quiescent and active neural stem cells (Llorente et al. 2022; Miranda‐Negrón and García‐Arrarás 2022).

As our meta‐analysis results reflect bulk dissections, these findings could imply functional changes within those cell types or altered cell type balance. To address this question, we conducted an exploratory follow‐up analysis comparing our meta‐analysis findings to snRNA‐Seq results characterizing the effects of FLX treatment (Nguyen, Sun, et al. 2025) and ECS (Santiago et al. 2026) on hippocampal cell types. Our findings showed a strong enrichment of antidepressant‐related expression amongst sets of genes previously shown to be differentially expressed in response to FLX and ECS in DG granule cells. Moreover, our meta‐analysis replicated previous results indicating that antidepressants (FLX, ECS) induce a transcriptional remodeling in DG granule neurons resembling a return to a developmental‐like state (Nguyen, Sun, et al. 2025; Santiago et al. 2026). To do this, antidepressants were theorized to reopen critical developmental periods by removing specific forms of the extracellular matrix (ECM), thereby allowing synaptic growth and plasticity (Nguyen, Sun, et al. 2025). Notably, we also observed differential expression of ECM‐related genes (e.g., *Aggrecan* (*Acan*) and *Heparan‐alpha‐glucosaminide N‐acetyltransferase* (*Hgsnat*)) previously shown to modulate anxiety and exploratory activity (Martins et al. 2015; Marcó et al. 2016), and an enrichment of antidepressant‐related effects in gene sets specific to fibroblasts, which secrete ECM components (collagen, proteoglycans) (Dick et al. 2025). These findings support the utility of triangulating between highly‐powered bulk analyses with diverse samples and smaller, but highly‐specific, single‐cell or spatial analyses.

### 4.2 Hippocampus: Differential Expression Related to Neurodevelopment and Morphogenesis

Many genes and pathways related to morphogenesis and neurodevelopment were also upregulated following antidepressant exposure, both within our hippocampal meta‐analyses and previous snRNA‐Seq studies (Nguyen, Sun, et al. 2025; Santiago et al. 2026). For example, *Plekha2* was the most strongly upregulated DEG in our meta‐analysis, and was previously found to be downregulated following chronic stress and upregulated in the DG following antidepressant interventions (FLX and exercise), correlating with increased AHN and dendritic spine density (Huang et al. 2012). *Plekha2* was also upregulated in DG granule neurons following ECS (Santiago et al. 2026), and encodes a protein (TAPP2) which promotes cell adhesion to the ECM (Rouillard et al. 2016; Diamant et al. 2025), suggesting a mechanism by which antidepressants might modulate ECM regulation of “immature‐like” and “mature‐like” states in DG granule cells. This protein also modulates a signaling axis (PI3K/AKT: (Li and Marshall 2015; Wullschleger et al. 2011; Landego et al. 2012)) proffered as a new antidepressant target (Chen et al. 2024) because it mediates neurogenic and growth factor (BDNF: (Yang et al. 2020)) effects in depression models (Wu et al. 2008; Jiang et al. 2021; Zheng et al. 2017; Bruel‐Jungerman et al. 2009). For these reasons, *Plekha2* is a promising candidate for follow‐up studies.

Several top DEGs have been directly tied to hippocampal morphology. For example, *Proliferation and apoptosis adaptor protein 15A* (*Pea15a*) was down‐regulated by antidepressants in our meta‐analysis and in DG granule neurons in response to ECS (Santiago et al. 2026) and encodes an abundant phosphoprotein modulating apoptosis and cell proliferation signaling (Park and Kang 2018; Araujo et al. 1993) that correlates with ECT‐induced hippocampal gray matter volume increases in depressed patients (Sun et al. 2024). *ALF transcription elongation factor 3 (Aff3)* was upregulated in our meta‐analysis and in multiple hippocampal cell types (DG neurons, astrocytes) following FLX or ECS treatment (Nguyen, Sun, et al. 2025; Santiago et al. 2026). It encodes a component of the transcriptional super elongation complex (AFF3/LAF4) important for the migration of neural progenitor cells (Moore et al. 2014). As loss of *Aff3* produces hippocampal atrophy and enlarged ventricles (Voisin et al. 2021), its upregulation could be important for antidepressant reversal of hippocampal atrophy. *Family With Sequence Similarity 135 Member B (Fam135b)* was also upregulated in our meta‐analysis and in hippocampal neurons following FLX or ECS treatment (Nguyen, Sun, et al. 2025; Santiago et al. 2026), and can promote neurite extension and neuron survival (Sheila et al. 2019), suggesting another mechanism for reversing depression‐related atrophy.

### 4.3 Hippocampus: Differential Expression Related to Neuronal Signaling and Neuroplasticity

Multiple genes responsible for neuronal signaling were downregulated by antidepressants in our meta‐analysis and in snRNA‐Seq datasets. For example, *Neurensin 1 (Nrsn1)* is a brain‐specific gene potentially important for neural plasticity and nerve signal transduction (Lencer et al. 2017; Cho et al. 2015) that is sensitive to chronic stress. *Nrsn1* was part of the enriched gene set signaling the return of DG granule neurons to a “developmental‐like” state theorized to be important for antidepressant effects (Nguyen, Sun, et al. 2025; Santiago et al. 2026). *Potassium Two Pore Domain Channel Subfamily K Member 10* (*Kcnk10*) was down‐regulated in our meta‐analysis, in DG granule neurons in response to ECS (Santiago et al. 2026), and following environmental enrichment, which has antidepressant‐like effects. It encodes potassium channels that can be stimulated by arachidonic acid, which is implicated in the development of depression (Regulska et al. 2020; Dong et al. 2015). *Unc‐13 homolog C* (*Unc13c*) was down‐regulated in our meta‐analysis and in DG granule neurons in response to FLX (Nguyen, Sun, et al. 2025) and mediates excitatory neurotransmission (Ansari et al. 2023), whereas another component of hippocampal glutamatergic synapses, *Syntaxin Binding Protein 5* (*Stxbp5*) (Barak et al. 2010; Batten et al. 2017) was upregulated. Collectively, these findings suggest molecular targets that may mediate antidepressant effects on neuroplasticity.

### 4.4 Hippocampus: Differential Expression Related to Stress Responses

Stress and its effects on the hippocampus are well‐documented in the pathophysiology of depression, with the hippocampus acting as both a target and regulator of stress (Dranovsky and Hen 2006). Several antidepressant DEGs regulate stress responses. For example, *T‐box Transcription Factor 19* (*Tbx19*) was upregulated by antidepressants and is an important regulator of the hypothalamus‐pituitary (HPA) stress‐response axis, correlating with neurotic traits (Wasserman et al. 2007). Moreover, sets of genes previously shown to respond to chronic stress were enriched with antidepressant differential expression, typically in the opposing direction. Individual antidepressant DEGs also showed stress‐opposing effects (e.g., *Plekha2*, *Uroplakin 1A (Upk1a)*), whereas other DEGs showed antidepressant effects mimicking chronic stress (e.g., *Nrsn1*, *GLIS family zinc finger 1* (*Glis1*), and corticosteroid‐sensitive *Cdk5 And Abl Enzyme Substrate 1* (*Cables1*) (Santiago et al. 2026; Gulyaeva 2019) and *MER proto‐oncogene tyrosine kinase* (*Mertk*) (Byun et al. 2023)). As some of these genes are tied to brain and behavior abnormalities following stress (Byun et al. 2023), these results may represent an activation of natural compensatory mechanisms. Our findings also suggest possible pathways for antidepressants to reverse stress‐effects on growth‐related processes, as *Cables1* is responsible for cell cycle progression (Gulyaeva 2019) and *Glis1* can promote the generation of pluripotent stem cells (Scoville et al. 2017).

### 4.5 Hippocampus: Differential Expression Related to Immune Function

Immune dysregulation is a prominent theory in depression (Miller and Raison 2016). Environmental stressors produce an increased immune response in the brain, including activation of hippocampal microglia (Price and Duman 2020; Calcia et al. 2016). Several classes of antidepressants, including SSRIs and SNRIs, modulate inflammatory mechanisms and oxidative stress (Mariani et al. 2022). We found that antidepressants upregulated many genes involved in immune cell recruitment, such as *C‐C motif chemokine receptor‐like 2* (*Ccrl2*), *Plekha2* (Landego et al. 2012), and *Guanylate Binding Protein 2 (Gbp2)* (Zhang et al. 2023). Many immune‐related cell types in the brain can release neurotrophic factors and anti‐inflammatory cytokines as well as pro‐inflammatory cytokines (Hu et al. 2015; Cope and Gould 2019), but the observed effects didn't clearly suggest a reduction in inflammation in favor of growth‐related processes. For example, *Gbp2* was upregulated by antidepressants, but promotes M1 (pro‐inflammatory) microglial polarization (You et al. 2024) and stress‐sensitive *Mertk* was down‐regulated and mediates anti‐inflammatory phagocytosis (Byun et al. 2023; Scott et al. 2001; Nguyen et al. 2023). Similarly, the enrichment of antidepressant effects in cytokine‐related gene sets could suggest pro‐inflammatory or anti‐inflammatory effects. *Toll‐like receptor 9* (*Tlr9*) was upregulated and can promote both, encouraging immune homeostasis (Matsuda et al. 2015; Hung et al. 2016).

### 4.6 Cortex: Differential Expression of a Gene Related to Microglial Function and Neuroplasticity

In the cortex, *Atp6v1b2* was the only significant DEG, upregulated across a variety of antidepressant treatments. However, it is a strong candidate for mediating antidepressant effects, as it contains a risk allele for depression (Shyn et al. 2011; Gonda et al. 2016), and is differentially methylated following antidepressant treatment (Barbu et al. 2022). *Atp6v1b2* encodes a component of vacuolar ATPase (V‐ATPase), a proton pump that acidifies intracellular organelles (Nishi and Forgac 2002) in a manner important for protein degradation, autophagy, small molecule transport, and receptor‐mediated endocytosis (Nishi and Forgac 2002; Collins and Forgac 2020). In microglia, *Atp6v1b2* is likely to promote phagocytosis and M2 anti‐inflammatory phenotype (Li et al. 2024; Fairley et al. 2023; Sharma et al. 2015). This could mediate antidepressant function, as microglial activation in the frontal cortex correlates with depression severity (Wang et al. 2022). V‐ATPase also plays an important role in neurons, generating the electrochemical gradient that facilitates neurotransmitter loading into vesicles (Gonda et al. 2016; Moriyama and Futai 1990; Eaton et al. 2021; Hinton et al. 2009; Egashira et al. 2015; Abbas et al. 2020), suggesting antidepressants could increase the efficiency of synaptic transmission. Furthermore, V‐ATPase can activate the mTOR pathway (Pamarthy et al. 2018), which may mediate rapid antidepressant effects (Li et al. 2010). Altogether, these findings suggest that *Atp6v1b2* is a promising candidate for follow‐up, along with other members of the V‐ATPase family (Filipović et al. 2022).

### 4.7 Limitations and Future Directions

#### 4.7.1 Regional Specificity

There was a positive correlation between the antidepressant effects identified in the hippocampus and cortex. Despite that overlap, there was a notable difference in the number of identified DEGs. This may indicate higher sensitivity and responsiveness in the hippocampus to antidepressants, perhaps conferred by the sensitivity of the DG and neurogenic niche, but could also reflect differences in statistical power (*n* = 313 vs. *n* = 233) and heterogeneity in the included subjects and tissue dissections. Notably, an exploratory meta‐regression combining data from both the hippocampus and cortex found minimal evidence that broadly defined brain region (hippocampus vs. cortex) was a primary source of antidepressant effect heterogeneity. However, variation in cortical region and cell type balance may still introduce heterogeneity in these bulk dissections. Within our cortical meta‐analysis, we included samples from multiple cortical regions, including the PFC and ACG (Pizzagalli and Roberts 2022). These regions are likely to serve different roles in the manifestation of depression symptoms and treatment response (Barazany and Assaf 2012; Jorstad et al. 2023). Exploratory analyses suggested that antidepressant effects in the ACG may differ from other cortical dissections, but it was difficult to draw strong conclusions because there were only 3 or fewer studies representing this region in the meta‐analysis for any particular gene. Future well‐powered studies will be needed to assess regional heterogeneity.

#### 4.7.2 Relevance to Human Clinical Populations

Future work should also explore the relevance of our findings to more diverse human clinical populations. Controlled rodent studies provide insight into causality, as treatment effects in clinical populations are often confounded by disease severity, but there are important species differences in brain physiology and morphology (Preuss and Wise 2022). Also, unlike clinical populations, our samples were almost all male, despite well‐documented sex differences in the neurobiological effects of depression in pre‐clinical and clinical studies (Eid et al. 2019).

Moreover, the scope of our search included datasets not derived from depression models, and antidepressants may affect neural structures and cognition in nondepressed subjects differently than depressed subjects (Willard et al. 2015; Prado et al. 2018), including in stress‐based animal models (Ibrahim et al. 2022). Notably, most cortical datasets used no depression model, which could account for weaker cortical antidepressant effects. To address this issue, we ran a meta‐regression that explored whether the inclusion of a depression model in a study (vs. only controls) modulated antidepressant effects. We found little impact, but our meta‐analysis project was not well‐designed to answer this question, as antidepressant effects were extracted from each study using the full sample and not subsetted by subject characteristics. Furthermore, the depression models represented in the meta‐analyses encompassed highly divergent pathways to depressive‐like behavior, including chronic stress, inflammation, and olfactory bulbectomy, which may be optimally reversed by different forms of treatment. Therefore, future studies are needed to better delineate differential treatment effects across animal depression models and subject demographics.

## 5 Conclusion

Altogether, our meta‐analyses identified 59 genes that were consistently differentially expressed following treatment with a variety of antidepressants, many of which were associated with functions previously implicated in depression. These genes and pathways are worth investigating as potential linchpins for antidepressant efficacy or targets for novel therapies, with triangulation of results across species to infer translatability. For future work, we plan to compare our results with environmental factors that alleviate depression, like environmental enrichment and exercise. Better‐powered studies should also follow up on our exploratory analyses examining how categories of antidepressants differ—instead of converge—in their effects to elucidate treatment resistance and identify predictors of individual response to different treatment categories.

## Author Contributions

**Mubashshir Ra'eed Bhuiyan:** conceptualization, methodology, writing – review and editing. **René Hen:** funding acquisition, writing – review and editing, supervision, conceptualization, methodology. **Erin Hernandez:** conceptualization, methodology, software, formal analysis, investigation, data curation, visualization, writing – original draft, writing – review and editing. **Adrienne N. Santiago:** writing – review and editing, data curation. **Stanley J. Watson Jr.:** writing – review and editing, funding acquisition. **Elizabeth I. Flandreau:** conceptualization, methodology, writing – review and editing. **Huda Akil:** writing – review and editing, funding acquisition, supervision. **Megan H. Hagenauer:** conceptualization, methodology, software, formal analysis, writing – review and editing, visualization, validation, funding acquisition, supervision, project administration. **Phi T. Nguyen:** writing – review and editing, data curation. **Sophie Mensch:** conceptualization, methodology, writing – review and editing. **Eva M. Geoghegan:** conceptualization, methodology, software, formal analysis, investigation, data curation, writing – review and editing, writing – original draft, visualization. **Sophia Espinoza:** conceptualization, methodology, software, data curation, formal analysis, investigation, writing – original draft, writing – review and editing, visualization.

## Funding

This work was supported by Hope for Depression Research Foundation, University of Michigan Undergraduate Research Opportunities Program, Grinnell College Center for Careers, Life, and Service, National Institute on Drug Abuse (NIDA U01 DA043098), and The Pritzker Neuropsychiatric Disorders Research Foundation.

## Conflicts of Interest

The authors declare no conflicts of interest. Several authors are members of the Pritzker Neuropsychiatric Disorders Research Consortium (M.H.H., H.A., S.J.W.), which is supported by the Pritzker Neuropsychiatric Disorders Research Fund L.L.C. A shared intellectual property agreement exists between this philanthropic fund and the University of Michigan, Stanford University, the Weill Medical College of Cornell University, the University of California at Irvine, and the HudsonAlpha Institute for Biotechnology to encourage the development of appropriate findings for research and clinical applications.

## Supporting information


**Figure S1:** Exploring heterogeneity in the antidepressant effects across hippocampal studies and contrasts.
**Figure S2:** Small sample size studies do not appear to be adding disproportionately to the noise in our hippocampal meta‐analysis.
**Figure S3:** Heatmap of the Top 50 Traditional Antidepressant Hippocampal Meta‐Analysis Genes Across Datasets.
**Figure S4:** Heatmap of the Top 50 Nontraditional Antidepressant Hippocampal Meta‐Analysis Genes Across Datasets.
**Figure S5:** Exploratory analysis: Traditional and nontraditional antidepressants have overlapping effects on the hippocampus.
**Figure S6:** A meta‐regression including antidepressant type (nontraditional vs. traditional) and dissection (dentate gyrus (DG) vs. whole hippocampus) as co‐variates provided insight into the heterogeneity in antidepressant effects observed in the hippocampal data.
**Figure S7:** A meta‐regression including antidepressant type (nontraditional vs. traditional) and dissection (dentate gyrus (DG) vs. whole hippocampus) as co‐variates produced estimates of antidepressant effects that closely resembled the estimates from our original meta‐analyses.
**Figure S8:** A meta‐regression including antidepressant type (nontraditional vs. traditional) and dissection (dentate gyrus (DG) vs. whole hippocampus) as co‐variates suggests that antidepressant effects are larger in the dentate gyrus.
**Figure S9:** Other potential hippocampal meta‐regression models produced less insight into the heterogeneity in antidepressant effects observed in the hippocampal data.
**Figure S10:** Heatmap of the Top 50 Cortical Meta‐Analysis Genes Across Antidepressant Datasets.
**Figure S11:** Exploring heterogeneity in the antidepressant effects across cortical studies and contrasts.
**Figure S12:** Small sample size studies do not appear to be adding disproportionately to the noise in our cortical meta‐analysis.
**Figure S13:** A cortical meta‐regression model including antidepressant type (nontraditional vs. traditional) and dissection (prefrontal cortex (PFC) vs. other cortex) as co‐variates produced minimal insight into the heterogeneity in antidepressant effects observed in the cortical data.
**Figure S14:** A meta‐regression including antidepressant type (nontraditional vs. traditional) and dissection (anterior cingulate (ACg) vs. other cortex) as co‐variates provides extremely tentative insight into the heterogeneity in antidepressant effects observed in the cortical data.
**Figure S15:** A meta‐regression including antidepressant type (nontraditional vs. traditional) and dissection (anterior cingulate (ACg) vs. other cortex) as co‐variates suggests that antidepressant effects are larger in the anterior cingulate.
**Figure S16:** A meta‐regression including both the hippocampal and cortical data, with brain region included as a co‐variate, identifies additional antidepressant‐related gene expression.
**Table S3:** Hippocampal meta‐analysis reveals genes that are consistently upregulated following antidepressant treatment (DEGs FDR < 0.05).
**Table S4:** Hippocampal meta‐analysis reveals genes that are consistently downregulated following antidepressant treatment (DEGs FDR < 0.05).
**Table S7:** 58 genes showed significant evidence of publication bias when considering the full hippocampal meta‐analysis output (Egger's test FDR < 0.05).
**Table S13:** A comparison of the FLX‐related hippocampal gene expression identified by the meta‐analyses in Ibrahim et al. (2022) and our antidepressant meta‐analysis results.
**Table S18:** Seven genes showed significant evidence of publication bias when considering the full cortical meta‐analysis output (Egger's test FDR < 0.05).

**Table S1:** The input used for both the hippocampal and cortical meta‐analyses.

**Table S2:** The full hippocampal antidepressant meta‐analysis results (16,494 genes, 16,439 stable meta‐analysis estimates).

**Table S5:** Hippocampal nondirectional fast Gene Set Enrichment Analysis (fGSEA) results (10,975 gene sets).

**Table S6:** Hippocampal directional fast Gene Set Enrichment Analysis (fGSEA) results (10,975 gene sets).

**Table S8:** Exploratory traditional antidepressant hippocampal meta‐analysis results (9,628 genes, 9,612 stable meta‐analysis estimates).

**Table S9:** Exploratory nontraditional antidepressant hippocampal meta‐analysis results (12,203 genes, 12,154 stable meta‐analysis estimates).

**Table S10:** Exploratory hippocampal results from a meta‐regression including antidepressant type (non‐traditional vs. traditional) and dissection (DG vs. whole hippocampus) as co‐variates (16,494 genes, 16,442 stable meta‐analysis estimates).

**Table S11:** Exploratory hippocampal results from a meta‐regression including antidepressant type (nontraditional vs. traditional), dissection (DG vs. whole hippocampus), and transcriptional profiling platform (microarray vs. RNA‐seq) as co‐variates (16,494 genes, 15,082 stable meta‐analysis estimates).

**Table S12:** Exploratory hippocampal results from a meta‐regression including antidepressant type (nontraditional vs. traditional), dissection (DG vs. whole hippocampus), and inclusion of a depression model (vs. control‐only) as co‐variates (16,494 genes, 16,148 stable meta‐analysis estimates).

**Table S14:** Hippocampal antidepressant meta‐analysis: Enrichment in gene sets derived from snRNA‐seq.

**Table S15:** The full cortical meta‐analysis results (15,583 genes, 15,454 stable meta‐analysis estimates).

**Table S16:** Cortical directional fast Gene Set Enrichment Analysis (fGSEA) results (10,579 gene sets).

**Table S17:** Cortical nondirectional fast Gene Set Enrichment Analysis (fGSEA) results (10,579 gene sets).

**Table S19:** Exploratory cortical results from a meta‐regression including antidepressant type (nontraditional vs. traditional) and dissection (PFC vs. other cortex) as co‐variates (15,583 genes, 15,406 stable meta‐analysis estimates).

**Table S20:** Exploratory cortical results from a meta‐regression including antidepressant type (nontraditional vs. traditional) and dissection (ACg vs. other cortex) as co‐variates (15,583 genes, 15,464 stable meta‐analysis estimates).

**Table S21:** Exploratory results from a meta‐regression encompassing both cortical and hippocampal datasets, with brain region included as a co‐variate (12,857 genes, 12,835 stable meta‐analysis estimates).

## Data Availability

This paper centers on secondary data analysis using publicly available datasets (see Table 1 for full list of accession numbers).
